# Exploring support needs of people living with diabetes during the coronavirus COVID-19 pandemic: insights from a UK survey

**DOI:** 10.1136/bmjdrc-2021-002162

**Published:** 2021-06-07

**Authors:** Sarah Sauchelli, Julia Bradley, Clare England, Aidan Searle, Alex Whitmarsh

**Affiliations:** National Institute for Health Research Bristol Biomedical Research Centre, University Hospitals of Bristol and Weston NHS Foundation Trust and University of Bristol, Bristol, UK

**Keywords:** disease management, COVID-19, healthcare surveys, health policy

## Abstract

**Introduction:**

The coronavirus COVID-19 pandemic has radically compromised healthcare for people living with chronic conditions such as diabetes. Government-imposed restrictions to contain the spread of the virus have forced people to suddenly adjust their lifestyle. This study aimed to capture the impact of the pandemic on people living with diabetes and the views of these individuals on ways in which the information, advice and support they are receiving could be improved.

**Research design and methods:**

An online anonymous survey was distributed across the UK during the first lockdown and initial easing. The survey comprised questions about confidence in diabetes self-management, resources used to obtain information, advice and support, and opinions on how these could be improved. Open-ended questions captured subjective experiences.

**Results:**

The survey was completed by 773 adults with diabetes (69.2% type 1, 28.5% type 2). There was notable variability in the impact of the pandemic on confidence in self-management, with confidence having deteriorated most commonly in the ability to take care of own mental well-being (37.0% respondents) and improved most commonly in maintaining a healthy weight (21.1% respondents). 41.2% of respondents living alone reported not receiving any outside support. The quality of information, advice and support received from the healthcare team was rated poorly by 37.2%. Respondents sought greater communication and tailored advice from their care team, clear and consistent information from the government and news channels, and improved understanding of diabetes and its challenges from their personal networks and employers.

**Conclusion:**

Adjusting to the COVID-19 pandemic has strained the mental health and well-being of people living with diabetes. Diabetes care teams must receive assistance to support these individuals without risking further inequalities in access to healthcare. Equipping personal networks and employers with knowledge on diabetes and skills to support self-management may reduce the burden on the National Health Service.

**Significance of this study**

**What is already known about this subject?**

- People living with diabetes mellitus, particularly those with poor blood glucose, are more vulnerable to developing the severe outcomes of COVID-19.
- National Health Service (NHS) prioritisation of COVID-19 has disrupted the availability of care for patients with chronic health conditions, including diabetes mellitus.

**What are the new findings?**

- The pandemic generated a decrease in confidence in diabetes self-management, particularly regarding mental well-being (37.0%) and adhering to physical activity recommendations (32.0%) and a healthy eating pattern (29.6%). Greater access to the healthcare team and services, strategies to adjust self-care (with greater focus on mental health) and more external support are deemed as important to reinstate diabetes self-management.
- Quality of information, advice and support received from the government and healthcare teams were perceived most poorly (respondents giving a rating of poor or very poor: 39.0% and 37.2%, respectively). There is a request for greater transparency, higher quality information, improved contact, and an increased understanding of the condition by others.

**How might these results change the focus of research or clinical practice?**

- A shift to remote consultations should include training practitioners to detect emotional distress in patients and the ability to refer patients to NHS or community-led mental health support.A collective effort is needed to produce more stratified and consistent guidance, with clear messaging to minimiseminimize uncertainty and distress.

## Introduction

The coronavirus COVID-19 is a severe respiratory syndrome generated by infection by SARS-CoV-2. On January 30, 2020, the WHO Emergency Committee declared COVID-19 a global health emergency,^1^ with approximately 41.77 million cases and 1.14 million deaths due to COVID-19 recorded worldwide within the first 10 months (https://ourworldindata.org/coronavirus). To contain the spread of the virus and protect the impact on the UK National Health Service (NHS), on March 23, 2020, the UK government imposed a national lockdown and the prioritisation of patients with COVID-19 across the NHS.^2^ From June 1, 2020, a range of physical distancing measures were imposed at varying degrees across time. Though these measures were useful for flattening the rate of infection, they caused severe disruption in the lives of people across the population,^3 4^ and in particular patient groups who rely on healthcare services.^5^

For people living with diabetes, COVID-19 prioritisation in the NHS caused severe disruptions to healthcare provision. This included the cancellation of routine check-up appointments (eg, glycated haemoglobin (HbA1c) and retinopathy checks), diabetes education sessions, and hospital services for non-urgent care. Additionally, support systems such as face-to-face peer support were suspended, while digitally delivered solutions were accelerated.^6^ As the pandemic persisted, NHS England published new guidelines encouraging a shift towards remote consultations whenever possible, the use of a case-by-case approach to evaluate the need for face-to-face reviews, and the uptake of digital self-management tools.^7^ In addition to practical challenges in rolling out these guidelines across the NHS, the success of these changes in care delivery relied on patients’ ability to adapt and engage in technology-assisted self-care, as well as practitioners’ ability to interpret data from technology and their confidence in delivering care via remote consultations.^8–10^

Given the nationally imposed restrictions and physical distancing policies, and the limited access to healthcare teams, we expected the pandemic would have a notable impact on everyday diabetes management and the mental health of people living with diabetes, their parents, carers, and partners. This study aimed to capture this impact and the views of these individuals on how to improve the information, advice and support they received during the pandemic.

## Research design and methods

An online survey was developed by the National Institute for Health Research Bristol Biomedical Research Centre (NIHR Bristol BRC) in collaboration with the Diabetes UK South West team. The first draft of the survey was developed based on questions posted on the Diabetes UK forum, Facebook diabetes support groups, and discussions with diabetes support teams (eg, Diabetes UK, Brigstowe) between April 1, 2020 and April 15, 2020. The first draft was reviewed by Diabetes UK volunteers to ensure language, structure and question appropriateness.

The survey comprised a mixture of multiple-choice questions to quantify events and compare answers across groups, and open questions to gain insight on individual experiences and opinions. This mixed-methods approach served to provide stakeholders with an overview of the impact that the pandemic has had on people living with diabetes, and subsequently draw out avenues for action guided by the people affected (ie, patient-led stakeholder decision-making). Responses were sought from people living with diabetes and their parents, carers and partners. Questions were adapted accordingly: parents, carers and partners were asked about their confidence in their ability to support diabetes self-management and their own experiences in obtaining information. The full survey, with all items and response options, can be seen in online supplemental file 1.

10.1136/bmjdrc-2021-002162.supp1 Supplementary data

### Outcome measures

- Demographic characteristics of the respondents, including diabetes type, postcode (first part only), age, gender, ethnicity, living situation.
- Information regarding the pandemic included physical distancing measures being taken at the time of completion (eg, following stringent physical distancing or shielding), diagnosis of COVID-19 or presence of symptoms, and changes in living circumstances due to COVID-19.
- Confidence in diabetes self-management was rated (Likert scale 0–10) across several components of self-care, from ‘could not do at all’ to 10 ‘certain could do’ before and during the pandemic.
- Impact of appointment cancellation and thoughts regarding what would help ameliorate diabetes self-management.
- Information was gathered on the resources used for guidance on physical distancing measures, general diabetes self-management, and support for emotional well-being.
- Respondents provided ratings (5-point Likert scales) on ease of access to information and support regarding the various aspects of diabetes self-management (‘very difficult’ to ‘very easy’), as well as the quality (‘very poor’ to ‘very good’) of the information, advice and support received from several sources (eg, government, Diabetes UK, healthcare team). When participants gave a ‘very poor’ or ‘poor’ rating, they were asked to provide their opinions on how to improve it.
- A final set of questions focused on the support received from respondents’ personal network.

Diabetes self-management was defined according to the National Institute for Health and Care Excellence recommendations^11^ and further revisions by CL, a dietitian with clinical expertise in diabetes care, and Diabetes UK volunteers: checking blood sugar, correcting for blood sugar, good understanding of blood glucose levels and how to regulate them, ability to select the correct foods to eat, maintaining a healthy weight, adhering to dietary and physical activity recommendations, and looking after emotional well-being (mental health). A final domain was added for some of the items, to reflect the specific steps people with diabetes are recommended to take if they experience COVID-19 symptoms (eg, checking for ketones).

The survey was distributed across the UK, between April 24, 2020 and the August 31, 2020. A convenience sample was recruited via dissemination of the survey by the networks of the NIHR Bristol BRC, the University of Bristol and Diabetes UK. Means of dissemination included research portals (eg, the Oxford University Hospitals NHS Foundation Trust), social media (eg, Facebook and Twitter), University of Bristol website, email contacts and monthly newsletters (eg, NIHR Bristol BRC and Diabetes UK). Participants were eligible for the study if they were aged 18 years or over, lived in the UK, and had either been diagnosed with diabetes or were the parent, carer, or partner of someone with diabetes.

Participants self-referred to the study by completing the survey and were not reimbursed for involvement. To ensure anonymity, participants were not asked to insert any identifiable personal information except for the first part of their postcode (to capture geographical area).

The data presented below reflect responses from people who identified themselves as living with diabetes. The number of respondents who were parents, carers, or partners of someone with diabetes was considered insufficiently large to draw conclusions (n=79). Results are nonetheless visible in online supplemental file 2.

10.1136/bmjdrc-2021-002162.supp2 Supplementary data

### Analysis

Summary statistics show participant responses to survey questions. Results are presented for all participants with diabetes and by the main diabetes types. For questions on confidence in diabetes self-management, data are presented using medians and IQRs. Differences in confidence scores before the pandemic and at survey completion were also calculated and participants were grouped by whether their scores decreased, were stable or increased.

Where multiple-choice questions included an ‘Other’ response, respondents were encouraged to expand on the answer. These were categorised by a single team researcher (JB) and agreement was sought with the principal investigator (SS). Where deemed more appropriate, a response was sorted into the pre-existing multiple-choice options (eg, ‘leaving the house only for exercise’ was classified as ‘adhering to physical/social distancing guidelines’).

Open-ended questions were analysed using an inductive thematic approach. The first 15 responses of open-ended items were reviewed independently by two researchers (SS and JB) to generate an initial codebook for each item. The codebook was further refined following discussion with AS and CE until consensus was reached. Code names were renamed to reflect data and identify themes. This approach led to the development of a definitive coding framework by which all responses were coded. Analysis was carried out using the NVivo V.12 software package. Given the required rapid turnaround of the work, the open-ended questions were split across the researchers (SS, JB, CE, AS), with two researchers independently reviewing a particular item. Coding and themes were then discussed as a group. For each theme, examples were selected and reported as quotes in the Results section, with participant diabetes type.

## Results

A total of 773 people living with diabetes responded (a further 79 participants were parents, partners, or carers of someone with diabetes). Though respondents were widely distributed across the UK, most came from the South East (n=193) and South West (n=142) regions of England.

Three peak response time points were identified in responses (June 24, July 20, and August 17). Response times matched (±2 days) major recruitment efforts but could not be linked to changes in government guidelines. Sample sizes were not sufficiently large to compare data across these time points, but the data can be seen in online supplemental file 3.

10.1136/bmjdrc-2021-002162.supp3 Supplementary data

### Demographic characteristics

Table 1 presents a breakdown of the demographic characteristics of respondents. Most were women (67.1%) and of white British ethnicity (90.1%). Mean age was of 47.9 (SD=14.5, range 18–80) years. A total of 69.2% of respondents reported living with type 1 diabetes mellitus (T1DM), 28.5% with type 2 diabetes mellitus (T2DM). Most respondents had not experienced symptoms of COVID-19 since the start of the pandemic (80.6%). The most common symptoms reported were coughing, shortness of breath, and fever. A total of 66.8% of respondents were adhering to government social/physical distancing guidelines stipulated at the time of survey completion, 9.8% were voluntarily shielding despite not having received explicit instructions.

**Table 1:** Demographic characteristics, COVID-19 symptoms and measures adopted by respondents with diabetes

	All (n=773)	Type 1 (n=535)	Type 2 (n=220)
Gender, n (%)			
Female	516 (67.1)	365 (68.6)	139 (63.5)
Male	249 (32.4)	165 (31.0)	78 (35.6)
Other	4 (0.5)	2 (0.4)	2 (0.9)
Age, mean (SD)	47.9 (14.5)	44.4 (14.2)	56.5 (11.4)
Ethnicity, n (%)			
Arab	1 (0.1)	1 (0.2)	0 (0.0)
Asian or Asian British: Chinese	3 (0.4)	0 (0.0)	3 (1.4)
Asian or Asian British: Indian	8 (1.0)	2 (0.4)	6 (2.7)
Asian or Asian British: Pakistani	1 (0.1)	1 (0.2)	0 (0.0)
Black or black British: Caribbean	4 (0.5)	0 (0.0)	4 (1.8)
Mixed: white and Asian	5 (0.7)	3 (0.6)	2 (0.9)
Mixed: white and black African	1 (0.1)	1 (0.2)	0 (0.0)
Mixed: white and black Caribbean	1 (0.1)	1 (0.2)	0 (0.0)
Other ethnic group	1 (0.1)	0 (0.0)	1 (0.5)
Other mixed background	1 (0.1)	1 (0.2)	0 (0.0)
Other white background	31 (4.0)	26 (4.9)	5 (2.3)
Prefer not to answer	3 (0.4)	2 (0.4)	1 (0.5)
White: British	693 (90.1)	485 (91.2)	192 (87.7)
White: Irish	16 (2.1)	9 (1.7)	5 (2.3)
Living circumstances, n (%)			
Living with others	649 (84.1)	458 (85.6)	176 (80.4)
Living alone	123 (15.9)	77 (14.4)	43 (19.6)
Symptoms of COVID-19; n (%)			
No	623 (81.0)	434 (81.8)	176 (80.0)
Yes	70 (9.1)	47 (8.9)	21 (9.5)
Diagnosis	2 (0.3)	2 (0.4)	0 (0.0)
Not sure	74 (9.6)	48 (9.0)	23 (10.5)
Physical/social distancing measures taken; n (%)			
Following stringent physical/social/physical distancing	513 (66.8)	355 (66.9)	147 (67.1)
Self-isolating at home	16 (2.1)	9 (1.7)	7 (3.3)
Shielding group	59 (7.7)	37 (7.0)	19 (8.7)
Shielding (but not in shielding group)	75 (9.8)	49 (9.2)	22 (10.0)
Key worker/still leaving home to work	97 (12.6)	75 (14.1)	22 (10.1)
Other	4 (5.7)	3 (0.6)	1 (0.5)
Don’t know	4 (0.5)	3 (0.6)	1 (0.5)

### Confidence in diabetes self-management

**Figure 1:**
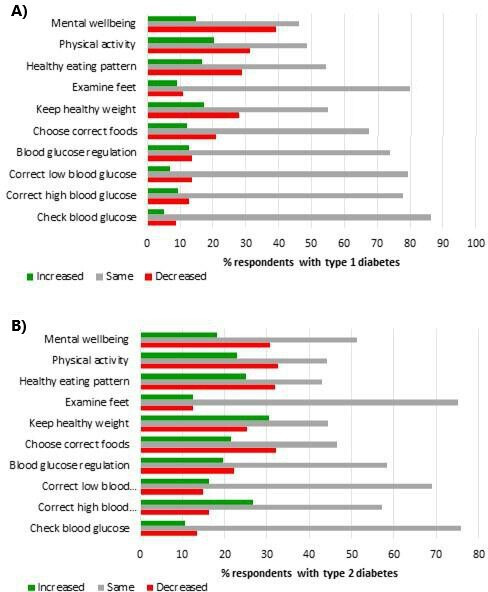
Change in confidence in diabetes self-management, derived from confidence at time of survey completion minus recall of confidence before the pandemic (n=770), for respondents with (A) type 1 diabetes, and (B) type 2 diabetes. Positive score (green): increase. Negative score (red): decrease.

Change in self-reported confidence in diabetes self-management was examined by comparing current confidence across various aspects of self-care with retrospective recall of confidence prior to the pandemic. Confidence in self-management was impacted more notably in the lifestyle components of diabetes self-management (eg, regular physical activity, healthy eating and maintenance of a healthy weight), and mental well-being (figure 1). Change in confidence was mainly negative (poorer), particularly for mental well-being (37% showed a decrease), though a proportion of respondents displayed improvements. No patterns were observed in changed confidence in diabetes self-management when comparing diabetes types (online supplemental file 2 for details).

Qualitative data analysis highlighted three main approaches (themes) through which respondents believed their confidence could be augmented: increased accessibility, adjusting self-care, and receipt of external support.

### Accessibility

Respondents indicated needing greater access to their care team and the support provided for diabetes self-management, greater opportunities for physical activity, and easier access to the food they need to adhere to dietary recommendations:

Lockdown limited exercise which I rely on to control sugar levels. Readjustment of insulin due to my exercise is not straight forward. (T1DM)

Several respondents indicated that receipt of blood testing tools would have facilitated diabetes self-management:

As a type 2 being able to monitor my blood sugar levels would be great but I have to rely on a six monthly check to see how I am doing. I did better when I bought my own monitor and strips but cannot afford £30+ per month to continue to do so. (T2DM)

Further, access to clearer guidance on individual risk was deemed important to facilitate decision-making:

Preparation guides for how to manage sugar levels if you get coronavirus. Also guidelines on how to stay vigilant as a diabetic when carrying out daily activities. (T1DM)

### Adjusting self-care

Respondents were aware that unhealthy habits may be attributed to their new circumstances generated by the pandemic:

Not working from home. Too close to the kitchen. (T2DM)

Respondents recognized the need to increase focus on mental health to reduce stress-induced glucose alterations:

My blood sugars have been more erratic due to the stress and worry for myself and my family, and they have been harder to keep under control. (T1DM)

Further, respondents recognized that this might require changes in doses or type of medication:

Reminders about changing insulin doses (via pump) in response to lower levels of physical activity. (T1DM)

### External support

Need for assistance from personal network and wider community was deemed important to increase confidence. This included support from family and friends, greater adherence to physical distancing from others, and help in household tasks and childcare:

Lack of help with childcare means difficulty in exercising and more strain at home, so sugars are harder to look after. (T1DM)

Outside of these three factors, several respondents indicated that resumption of ‘normal’ life would be needed:

Once things get back to normal and I can get back to my routine. (T2DM)

### Consequences of canceled appointments

This domain explored the impact of disruption in healthcare provision; by capturing how many respondents were affected and how they were affected. A total of 53.3% of T1DM and 46.4% of T2DM respondents had at least one appointment canceled at the time of survey completion. Qualitative analysis revealed four themes reflecting the type of issues faced by respondents due to the cancellation of appointments: lack of knowledge and confidence, difficulties in switching treatment, mental health, and empowerment in self-management.

### Lack of knowledge and confidence

Cancellation of appointments resulted in uncertainty on glucose control, difficulties in interpreting information provided by monitoring devices, and lack of confidence in the actions to take to improve glucose control:

My self-confidence has plunged, and lack of follow-up hasn’t helped. The clinic canceled appointments and I didn’t know who else to consult. (T1DM)

I have given up. I just pretend I do not have diabetes. (T2DM)

### Difficulties in switching treatment

Respondents indicated struggling to switch to other medications or changing doses and receiving adequate support to do so. They have had difficulties in using remote medical care, and experienced delayed or canceled referrals to other services:

I was on a pathway of improving my treatment methods (a pump) but that has been paused. (T1DM)

### Mental health

Reduced support and advice regarding self-management or risk, and the cancellation of appointments were posing a strain on respondents’ mental health and motivation to continue self-management:

Although I don’t feel less able to self-manage, I have sometimes felt less motivated to manage my diabetes well. A result of general anxiety and poor sleep. (T1DM)

### Empowerment in self-management

A few respondents indicated that they had managed to adapt to circumstances to improve self-management:

I have had to learn to cope and have read more and joined a Facebook diabetes support group, run by other diabetics. (T2DM)

### Ease of access to information, advice and support

This domain captured the degree of difficulty respondents experienced, from their viewpoint, to receive information, advice, and support regarding diabetes management, particularly in the context of COVID-19. Overall, people with T2DM made less use of the range of external resources available for information, advice, and support (including websites, healthcare teams, personal network and employer). For both diabetes types, the resources rated as most used were news channels (T1DM: 46.1%, T2DM: 52.8%), the public health and government website (T1DM: 12.5%, T2DM: 13.9%), and Diabetes UK (T1DM: 15.7%, T2DM: 13%) (see online supplemental file 2 for details).

**Figure 2:**
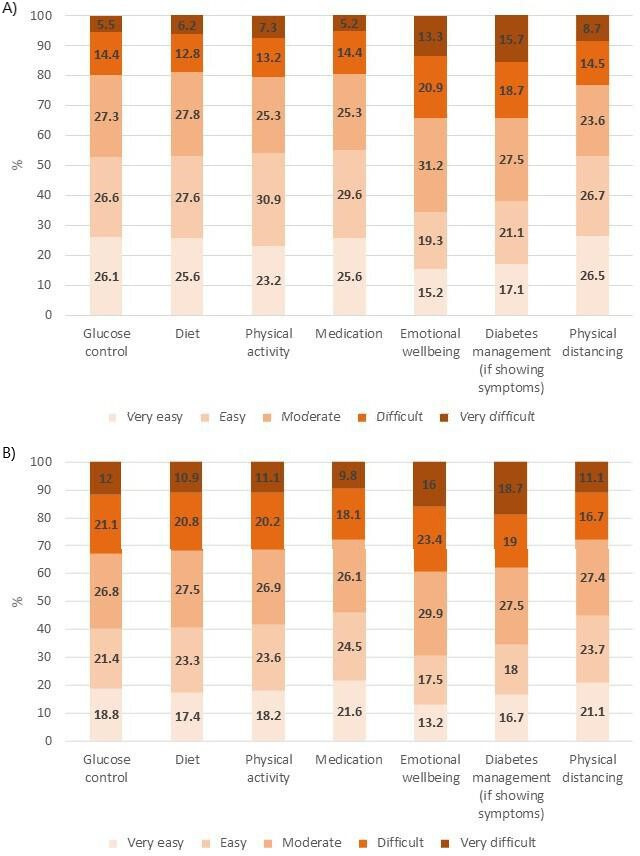
Rated difficulty in accessing (A) information and advice, and (B) support across diabetes self-management domains and adherence to physical distancing guidelines.

Respondents found it harder to receive support compared with information and advice. Access was more likely to be rated as ‘difficult’ or ‘very difficult’ in the domains ‘emotional well-being’ and ‘diabetes management if showing symptoms of COVID-19’ (figure 2). There were clear differences between diabetes types in access to support: 42.5% of respondents with T2DM reported ‘difficult’ or ‘very difficult’ access to support for glucose control, compared with 28.9% of respondents with T1DM. Among those respondents who reported living alone, 41.2% indicated that they were not receiving support from outside the household. External support was received primarily from the family (68.7%), friends (67.2%) and neighbors (28.4%).

### Perceived quality of information, advice and support

In this domain, respondents were asked to rate the quality (from ‘very poor’ to ‘very good’) of the information, advice and support received from various sources, ranging from social media to the healthcare team. Respondents who had provided ‘poor’ or ‘very poor’ scores were asked to suggest improvements that could be made. These qualitative data were purposely sought to assist stakeholders prioritize actions to be taken from the viewpoint of beneficiaries.

**Figure 3:**
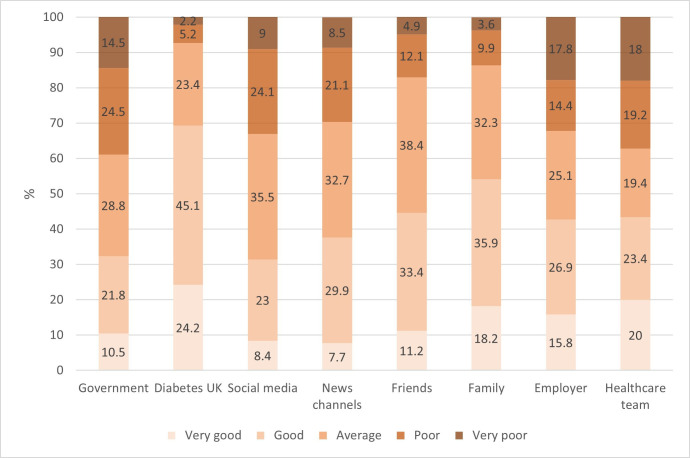
Reported quality of information, advice and support received from various resources.

Figure 3 shows respondents’ views on the quality of information, advice and support available across a wide range of sources. A total of 39.0% of respondents rated the quality of government guidance and support as ‘poor’ or ‘very poor’, with lower scores from T1DM (41.8%) than T2DM (31.7%) (online supplemental file 2). Perceived quality in the guidance and support received from healthcare teams was similar, with 37% of respondents considering it as ‘poor’ or ‘very poor’. In this case, ratings were poorer from T2DM (43.2%) compared with T1DM respondents (35.2%). No other patterns were observed between diabetes types.

**Figure 4:**
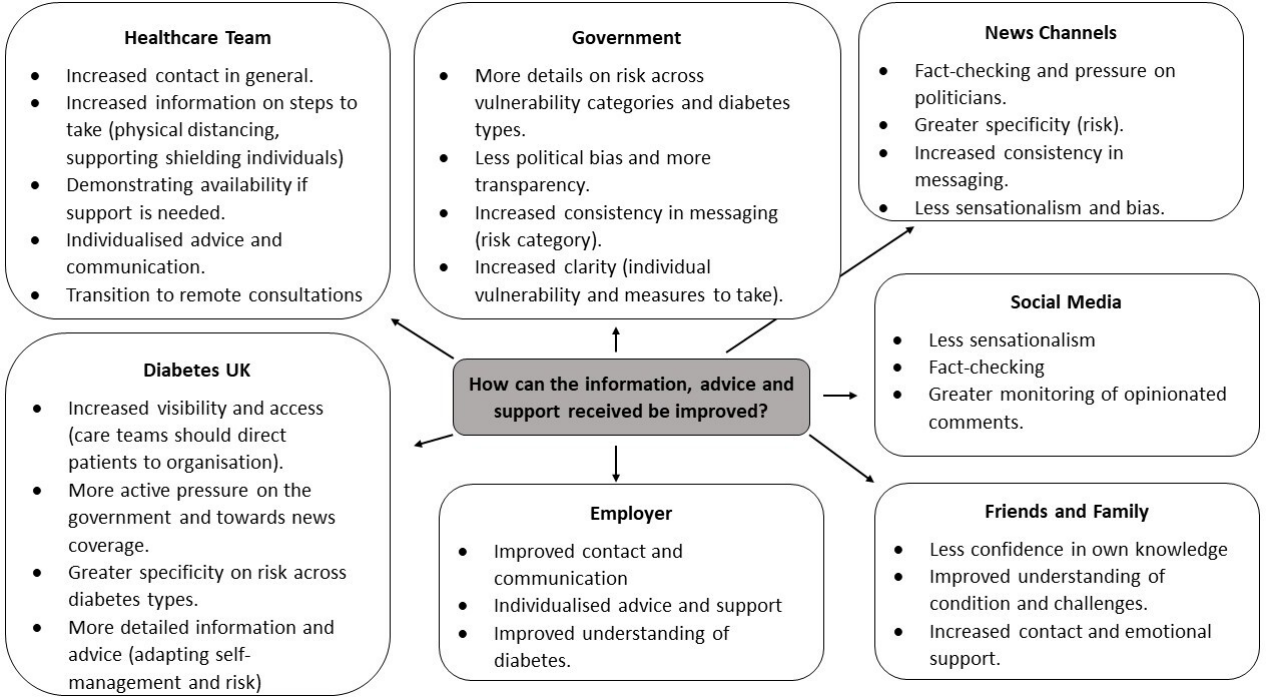
Main categories that emerged in respondents’ recommendations for improvement presented.

Figure 4 displays the main categories that emerged from the qualitative analysis, subdivided according to source queried. Four overarching themes were revealed: greater transparency, higher quality information and improved contact, and greater understanding of the condition.

### Greater transparency

Respondents expressed concerns regarding bias and tendency towards sensationalism in the information from the government, news channels and social media:

They over emphasise the negatives and cause fear or anxiety. (T2DM, news channels)

They requested these sources be more transparent in the evidence behind information and decision-making, greater fact-checking, objective reporting, and pressure on politicians to provide accurate information:

More challenge of government when information is inconsistent or ambiguous. (T1DM, news channels)

Fake news and anti-vac messaging to be removed promptly. (T1DM, social media)

It would be better if it came across as completely open and trustworthy. (T1DM, government)

### Higher quality information

Respondents also communicated the need for improvement in information provided by healthcare teams, government, Diabetes UK, news channels and employers. They requested more information on precautionary measures to take in terms of shielding/physical distancing, how the personal network can help in emergencies, and diabetes self-management:

Needs more clarity for people like me who are ‘vulnerable’ but have not received the NHS letter. (T2DM, government)

Got told I had to return to work, no discussion about how worried that made me. (T1DM, employers)

When I had a hypo and was very mixed up and no one in the family intervened because of us being distanced inside the home. (T1DM, healthcare team)

Data revealed that specificity was a frequent priority for improving the quality of information, distinguishing people with diabetes from other vulnerable people and differentiating between diabetes types. Greater specificity was sought for information on risk and for guidance on diabetes self-management:

Most of the dietary advice seems more geared to type 1 and doesn’t help me to lower my type 2 blood glucose. (T2DM, Diabetes UK)

Explain what the relevance of vulnerability to C-19 is in relation to what types of diabetics (type 1 or 2), those with complications etc, not just say ‘diabetics’. (T1DM, news channels)

No specific policy for diabetics. Only general advice for people more vulnerable. (T1DM, employer)

Consistency in the information provided was also deemed important:

Changing risk category of Diabetes since the beginning. Caused lots of confusion. (T1DM, government)

Several respondents, however, communicated that they had noticed improvements with time:

The information was much more clear. Particularly as they spoke about T1 and T2 separately. (T1DM, Diabetes UK)

### Improved contact and communication

Respondents frequently reported absence of their healthcare teams and employers, which had a negative impact on their mental health:

No contact from manager at this time and waiting for information has made this time more stressful. (T2DM, employer)

I have not received any information at all from my diabetes health care team. (T2DM, healthcare team)

There was a request for individualized contact and for the healthcare team to demonstrate availability if urgent support was needed:

I do feel that a quick phone call or more personal email would have been good. (T1DM, healthcare team)

I have contacted my diabetic nurse several times, the only reply I have received is a text message suggesting I go to diabetes UK website. (T2DM, healthcare team)

Would be good to hear more of “please contact us if there is a problem” rather than always “stay away from the surgery.” (T1DM, healthcare team)

Opinions regarding the support provided by healthcare teams varied across respondents, as some indicated that their care team was responsive:

Rang me to check I was ok as check-up delayed. Could ring if I wanted to. (T2DM, healthcare team)

Several respondents expressed an interest in remote consultations if this increased contact with their care team:

Improve access to diabetes team by telephone. (T2DM, healthcare team)

### Increased understanding of diabetes

Respondents expressed wanting their personal networks and employers to have a better understanding of their condition and the challenges faced:

Unless you have an illness and keep being told about having a underlying illness is harmful during this time, you just don't understand. (T2DM, friends and family)

It would be good if they were a little better informed, particularly, now, about the increased risks posed to people with diabetes by Covid-19. (T1DM, employer)

This was important to enhance experienced support:

Friends are a very important source of general support. (T1DM, friends and family)

## Conclusions

This study provides valuable insight in the ways people living with diabetes have been impacted by the coronavirus COVID-19 pandemic. As expected, NHS prioritisation of COVID-19 has had a negative impact on the access and level of support most people with diabetes have had during the pandemic, as experienced by people living with other chronic conditions.^12^ Closure of sporting facilities and home confinement have contributed to a reduced exercise, adoption of unhealthy dietary habits and weight gain in people with diabetes.^13^ This reflects respondents’ decreased confidence in self-management in these domains.

Reported difficulties in diabetes self-management are concerning given widespread evidence that people with diabetes, particularly those with comorbid obesity and poor blood glucose control, are at increased likelihood of hospital admission and negative outcomes from COVID-19.^14 15^ Research shows that a balanced diet can have a positive effect for prevention and management of COVID-19 in patients with diabetes.^16^ Though, alike seen in the general population,^17^ some respondents reported increased confidence in diabetes self-management, challenges to the ability to adhere to dietary and physical activity recommendations can worsen outcomes from COVID-19 through weight gain and glucose deregulation.

Initial results of steps taken to support self-management during the pandemic are emerging. A switch to remote consultations, delivered either via phone or video calls, during strict lockdowns has been linked with reduced HbA1c.^18^ Similarly, pairing flash glucose monitoring with remote control has shown promising outcomes.^19^ However, discrepancies among healthcare systems across countries must be taken into account. For example, in the UK the large majority (around 90%) of people with diabetes are managed by primary care,^20^ enabling healthcare teams to be key players in the provision of information and support remotely, but people with T2DM are not normally prescribed continuous glucose monitoring kits. The lack of universal care coverage in the USA requires decisive action from the government and stakeholders to increase accessibility to self-management support and medication.^20^

The disparities across respondents of this survey in the contact they had with healthcare teams highlights another obstacle to be addressed by health commissioners: ensuring equitable access to remote care. Negligible differences were found between diabetes types in ratings of quality of information, advice, and support from care teams, despite people with T1DM normally having considerably more contact with their care team than those with T2DM. Difficulties in accessing healthcare teams may be linked to practice-level differences in availability and capacity to shift to remote care. A centralized effort is therefore required to provide adequate resources and training for care teams to successfully make this transition.

Professional organizations can additionally work collaboratively to generate alternative avenues through which people can receive advice and support. For example, the Italian Society of Diabetes and the Association of Italian Diabetologists have partnered to give people with diabetes and their relatives direct access to specialists via a social media platform.^21^ These initiatives could help mitigate some of the impact of canceled appointments reported by respondents, for example, by providing expert advice regarding glucose monitoring, adjusting medication, and recommendations to improve glucose control.

Findings from this study also emphasise the need to augment opportunities for people with diabetes to obtain mental health support; respondents reported a loss of confidence in taking care of own mental well-being and difficulties accessing support in this domain. Findings resonate with research demonstrating an increase in psychiatric disorders and diabetes-related emotional distress during COVID-19.^22^ This is concerning in light of evidence showing that people with poorer psychological well-being were more likely to show a reduction in HbA1c and body mass index during lockdown.^23^

Organizations representing people living with diabetes have already taken steps to facilitate access to ongoing support by assisting people shift to online solutions.^6^ This may be an avenue to connect people living alone with the community for external support and reduce isolation, which is a primary contributor to mental health difficulties.^24^ Further, equipping the personal network with an increased understanding of diabetes and its challenges was also seen as important to increase the quality of support received. This aligns with extensive work demonstrating the value of a supportive immediate environment for the management of diabetes and well-being.^25^

Respondents of this survey additionally called for the implementation of policies to minimize sensationalism, misinformation, and improved communication between stakeholders and people living with diabetes. A collective effort is therefore required, focusing on stratified and consistent guidance on individual vulnerability, on how to self-manage diabetes while minimizing risk, and ensuring that people feel they can trust the entity communicating the information. Though greater communication and transparency have been greatly demanded throughout the pandemic,^26^ this study further shows how clear messaging is crucial to make vulnerable individuals feel safe in uncertain circumstances.

Some methodological limitations need to be taken into consideration. The survey was distributed online, meaning that participants would have a degree of digital literacy. The survey may not accurately capture the views of individuals who engage less with healthcare teams or their community, and we did not reach people who are unable to access technology. Though multimodal steps were taken to raise awareness of the survey, ethnic minorities and men were under-represented. Alternative strategies should be adopted to target these groups, especially as the prevalence of diabetes is elevated in ethnic minority communities.^27^ Further, though the healthcare environment did not change greatly from April until August 2020, this study was not powered to measure the individual impact of specific changes in guidance and messaging from the government and media during this period. These limitations were in part due to the urgency of distributing the survey for Diabetes UK to take timely action, and obstacles faced due to the pandemic in engaging with key people who could facilitate wider participation.

Despite its limitations, this study provides important insight into how the coronavirus COVID-19 pandemic has impacted people living with diabetes and their views on opportunities for improvement. As routine care is being canceled due to increased infection rates and the roll out of vaccines, it is essential that experiences and opinions from the initial wave of the pandemic are incorporated in stakeholder decision-making. As the pandemic has generated a transition to digital solutions to provide information, advice and support, efforts should also be made to ensure people less familiar with technology are not excluded. Development of these solutions should be adapted to the expected technology proficiency of the target group, available in multiple languages and accommodate for physical or mental disabilities.^8^ Alternative solutions should be provided for those from lower economic backgrounds or with limited access to internet.

## Data Availability

Anonymized data are available upon reasonable request.
