# Supplementary material for: Exploring support needs of people living with diabetes during the coronavirus COVID-19 pandemic: insights from a UK survey
Source: BMJ Open Diabetes Res Care. 2021 Jun 7;9(1):e002162. doi: 10.1136/bmjdrc-2021-002162 (PMC8186741; doi:10.1136/bmjdrc-2021-002162)
Supplement: Supplementary data [file bmjdrc-2021-002162supp002.pdf]

Supplementary file 2: support needs of people living with diabetes during COVID-19

Supplementary File 2

Title: Addressing support needs of people living with diabetes during the coronavirus COVID-19 pandemic: insights from a UK survey.

Sarah Sauchelli*[1], Julia Bradley[1], Clare England[1], Aidan Searle[1], Alex Whitmarsh[1]

[1] *National Institute for Health Research Bristol Biomedical Research Centre, University Hospitals of Bristol and Weston NHS Foundation Trust and University of Bristol.*

Responses to the survey

1. Responses from people living with diabetes

1.1. Geographical distribution of responses

**Region/Nation**	**n**	**%**
Scotland	89	11.6
Wales	32	4.18
East Midlands	31	4.05
East of England	52	6.8
Greater London	65	8.5
North East	14	1.83
North West	55	7.19
Northern Ireland	14	1.83
South East	193	25.2
South West	142	18.6
West Midlands	57	7.45
Yorkshire & Humber	21	2.75
**Total**	**765**	

1.2. Demographic characteristics.

**Diabetes group**	**n**	**%**
Type 1 diabetes	535	69.2
Type 2 diabetes	220	28.5
Another type of diabetes	18	2.3
	773	100.0

Supplementary file 2: support needs of people living with diabetes during COVID-19

	All (n=773)	Type 1 (n=535)	Type 2 (n=220)
**Gender, n (%)**			
Female	516 (67.1%)	365 (68.6%)	139 (63.5%)
Male	249 (32.4%)	165 (31.0%)	78 (35.6%)
Other	4 (0.5%)	2 (0.4%)	2 (0.9%)
**Age, mean (SD)**	47.9 (14.5)	44.4 (14.2)	56.5 (11.4)
**Ethnicity, n (%)**			
Arab	1 (0.1%)	1 (0.2%)	0 (0.0%)
Asian or Asian British: Chinese	3 (0.4%)	0 (0.0%)	3 (1.4%)
Asian or Asian British: Indian	8 (1.0%)	2 (0.4%)	6 (2.7%)
Asian or Asian British: Pakistani	1 (0.1%)	1 (0.2%)	0 (0.0%)
Black or Black British: Caribbean	4 (0.5%)	0 (0.0%)	4 (1.8%)
Mixed: White and Asian	5 (0.7%)	3 (0.6%)	2 (0.9%)
Mixed: White and Black African	1 (0.1%)	1 (0.2%)	0 (0.0%)
Mixed: White and Black Caribbean	1 (0.1%)	1 (0.2%)	0 (0.0%)
Other ethnic group	1 (0.1%)	0 (0.0%)	1 (0.5%)
Other Mixed background	1 (0.1%)	1 (0.2%)	0 (0.0%)
Other White background	31 (4.0%)	26 (4.9%)	5 (2.3%)
Prefer not to answer	3 (0.4%)	2 (0.4%)	1 (0.5%)
White: British	693 (90.1%)	485 (91.2%)	192 (87.7%)
White: Irish	16 (2.1%)	9 (1.7%)	5 (2.3%)

Living circumstances

**Are you currently living alone?**	All	Type 1	Type 2
No	649 (84.1%)	458 (85.6%)	176 (80.4%)
Yes	123 (15.9%)	77 (14.4%)	43 (19.6%)
	772 (100.0%)	535 (100.0%)	219 (100.0%)

**Has the number of people you are living with changed as a result of the coronavirus pandemic?**	All	Type 1	Type 2
No	684 (88.9%)	467 (87.6%)	200 (91.7%)
Yes	85 (11.1%)	66 (12.4%)	18 (8.3%)
	769 (100.0%)	533 (100.0%)	218 (100.0%)

Sauchelli S, et al. BMJ Open Diab Res Care 2021; 9:e002162. doi: 10.1136/bmjdrc-2021-002162

Supplementary file 2: support needs of people living with diabetes during COVID-19

Circumstances in relation to COVID-19

**Have you been diagnosed with or displayed symptoms of coronavirus since the beginning of February?**	**All**	**Type 1**	**Type 2**
No	623 (81.0%)	434 (81.8%)	176 (80.0%)
Yes	70 (9.1%)	47 (8.9%)	21 (9.5%)
Diagnosed with coronavirus	2 (0.3%)	2 (0.4%)	0 (0.0%)
Not sure	74 (9.6%)	48 (9.0%)	23 (10.5%)
	769 (100.0%)	531 (100.0%)	220 (100.0%)

**Which of the following best describes your current circumstances?**	**All**	**Type 1**	**Type 2**
Following stringent Physical/social/physical distancing	513 (66.8%)	355 (66.9%)	147 (67.1%)
Self-isolating at home	16 (2.1%)	9 (1.7%)	7 (3.3%)
Shielding group	59 (7.7%)	37 (7.0%)	19 (8.7%)
Shielding (but not in shielding group)	75 (9.8%)	49 (9.2%)	22 (10.0%)
Key worker/still leaving home to work	97 (12.6%)	75 (14.1%)	22 (10.1%)
Other	4 (5.7%)	3 (0.6%)	1 (0.5%)
Don't know	4 (0.5%)	3 (0.6%)	1 (0.5%)
	768 (100.0%)	531 (100.0%)	219 (100.0%)

1.3. Confidence in diabetes self-management

**BEFORE** the coronavirus pandemic and social/physical distancing guidance I was confident that I was able to…	**All**		**Type 1**		**Type 2**	
	n	Median (IQR)	n	Median (IQR)	n	Median (IQR)
Check blood glucose	720	10 (10, 10)	528	10 (10, 10)	174	10 (9, 10)
Correct high blood glucose	703	10 (8, 10)	530	10 (9, 10)	156	7 (3, 10)
Correct low blood glucose	680	10 (9, 10)	526	10 (10, 10)	136	10 (7, 10)
Good blood glucose regulation	571	9 (7, 10)	363	10 (8, 10)	197	8 (5, 10)
Choose correct foods	729	10 (8, 10)	519	10 (9, 10)	192	8 (5, 10)
Keep healthy weight	755	7 (4, 10)	525	8 (5, 10)	212	5 (2, 8)
Examine feet	769	10 (8, 10)	533	10 (8, 10)	218	10 (7, 10)
Healthy eating pattern	770	8 (6, 10)	534	9 (7, 10)	218	7 (5, 9)
Physical activity	770	8 (5, 10)	534	8 (6, 10)	218	6 (4, 9)
Mental wellbeing	770	8 (5, 10)	534	8 (6, 10)	218	7 (5, 10)

Note: score given on a Likert scale ranging from 0 (Could not do at all) to 10 (Certain could do). Not applicable was also an option to account inter-individual variability in condition and self-management requirements.

**AT PRESENT**, I am confident that…	**All**		**Type 1**		**Type 2**	
	n	Median (IQR)	N	Median (IQR)	n	Median (IQR)
Check blood glucose	727	10 (10, 10)	530	10 (10, 10)	179	10 (9, 10)
Correct high blood glucose	718	10 (8, 10)	531	10 (9, 10)	170	7 (4, 10)
Correct low blood glucose	697	10 (9, 10)	526	10 (9, 10)	153	9 (6, 10)
Good blood glucose	578	9 (7, 10)	367	10 (8, 10)	200	8 (5, 10)

Supplementary file 2: support needs of people living with diabetes during COVID-19

regulation						
Choose correct foods	744	9 (7, 10)	524	10 (8, 10)	202	8 (5, 10)
Keep healthy weight	759	7 (4, 9)	524	7 (5, 10)	217	5 (2, 7)
Examine feet	764	10 (8, 10)	527	10 (8, 10)	219	10 (7, 10)
Healthy eating pattern	766	8 (6, 10)	530	8 (6, 10)	218	7 (5, 9)
Physical activity	768	7 (5, 10)	531	8 (5, 10)	219	6 (4, 8)
Mental wellbeing	765	7 (5, 9)	530	7 (5, 9)	217	7 (4, 9)

Note: score given on a Likert scale ranging from 0 (Could not do at all) to 10 (Certain could do). Not applicable was also an option to account inter-individual variability in condition and self-management requirements.

**Change in score**	**All**	**Type 1**	**Type 2**
**Check blood glucose**			
Decreased	68 (9.6%)	45 (8.6%)	23 (13.5%)
Same	600 (84.3%)	453 (86.5%)	129 (75.9%)
Increased	44 (6.2%)	26 (5.0%)	18 (10.6%)
**Correct high blood glucose**			
Decreased	95 (13.6%)	67 (12.7%)	25 (16.2%)
Same	510 (73.1%)	410 (77.8%)	88 (57.1%)
Increased	93 (13.3%)	50 (9.5%)	41 (26.6%)
**Correct low blood glucose**			
Decreased	92 (13.7%)	71 (13.7%)	20 (14.8%)
Same	522 (77.7%)	412 (79.4%)	93 (68.9%)
Increased	58 (8.6%)	36 (6.9%)	22 (16.3%)
**Good blood glucose regulation**			
Decreased	92 (16.6%)	48 (13.5%)	42 (22.1%)
Same	380 (68.5%)	262 (73.8%)	111 (58.4%)
Increased	83 (15.0%)	45 (12.7%)	37 (19.5%)
**Choose correct foods**			
Decreased	173 (24.1%)	107 (20.8%)	60 (32.1%)
Same	440 (61.2%)	345 (67.1%)	87 (46.5%)
Increased	106 (14.7%)	62 (12.1%)	40 (21.4%)
**Keep healthy weight**			
Decreased	202 (27.1%)	144 (27.9%)	53 (25.2%)
Same	386 (51.8%)	284 (54.9%)	93 (44.3%)
Increased	157 (21.1%)	89 (17.2%)	64 (30.5%)
**Examine feet**			
Decreased	87 (11.4%)	58 (11.0%)	27 (12.4%)
Same	598 (78.7%)	419 (79.8%)	163 (75.1%)
Increased	75 (9.9%)	48 (9.1%)	27 (12.4%)
**Healthy eating pattern**			
Decreased	226 (29.6%)	153 (28.9%)	69 (31.9%)
Same	393 (51.5%)	287 (54.3%)	93 (43.1%)
Increased	144 (18.9%)	89 (16.8%)	54 (25.0%)
**Physical activity**			
Decreased	245 (32.0%)	166 (31.3%)	71 (32.7%)
Same	361 (47.2%)	257 (48.5%)	96 (44.2%)
Increased	159 (20.8%)	107 (20.2%)	50 (23.0%)
**Mental wellbeing**			

Supplementary file 2: support needs of people living with diabetes during COVID-19

Decreased	282 (37.0%)	207 (39.1%)	66 (30.7%)
Same	362 (47.5%)	244 (46.1%)	110 (51.2%)
Increased	118 (15.5%)	78 (14.7%)	39 (18.1%)

Difference between respondents living alone and those living with others

Change in score	Not living alone	Living alone
**Check blood glucose**		
Decreased	55 (9.0%)	13 (12.6%)
Same	516 (84.9%)	84 (81.6%)
Increased	37 (6.1%)	6 (5.8%)
**Correct high blood glucose**		
Decreased	71 (11.9%)	24 (23.3%)
Same	445 (74.8%)	65 (63.1%)
Increased	79 (13.3%)	14 (13.6%)
**Correct low blood glucose**		
Decreased	70 (12.3%)	22 (21.8%)
Same	451 (79.0%)	71 (70.3%)
Increased	50 (8.8%)	8 (7.9%)
**Good blood glucose regulation**		
Decreased	74 (15.6%)	18 (22.0%)
Same	327 (69.1%)	53 (64.6%)
Increased	72 (15.2%)	11 (13.4%)
**Choose correct foods**		
Decreased	150 (24.6%)	23 (20.9%)
Same	373 (61.2%)	67 (60.9%)
Increased	86 (14.1%)	20 (18.2%)
**Keep healthy weight**		
Decreased	166 (26.3%)	36 (31.6%)
Same	329 (52.2%)	57 (50.0%)
Increased	135 (21.4%)	21 (18.4%)
**Examine feet**		
Decreased	68 (10.6%)	19 (16.1%)
Same	508 (79.3%)	90 (76.3%)
Increased	65 (10.1%)	9 (7.6%)
**Healthy eating pattern**		
Decreased	185 (28.7%)	41 (35.0%)
Same	335 (51.9%)	57 (48.7%)
Increased	125 (19.4%)	19 (16.2%)
**Physical activity**		
Decreased	190 (29.5%)	55 (46.2%)
Same	309 (47.9%)	51 (42.9%)
Increased	146 (22.6%)	13 (10.9%)
**Mental wellbeing**		
Decreased	235 (36.6%)	47 (39.5%)
Same	301 (46.9%)	60 (50.4%)
Increased	106 (16.5%)	12 (10.1%)

Supplementary file 2: support needs of people living with diabetes during COVID-19

Differences between individuals living alone that were not receiving outside support and those that were.

Change in score	Not receiving outside support	Receiving outside support
**Check blood glucose**		
Decreased	7 (17%)	5 (9%)
Same	30 (73%)	51 (89%)
Increased	4 (10%)	1 (2%)
**Correct high blood glucose**		
Decreased	10 (25%)	14 (24%)
Same	23 (57%)	39 (66%)
Increased	7 (18%)	6 (10%)
**Correct low blood glucose**		
Decreased	6 (16%)	15 (26%)
Same	30 (79%)	38 (66%)
Increased	2 (5%)	5 (9%)
**Good blood glucose regulation**		
Decreased	7 (22%)	10 (22%)
Same	19 (59%)	33 (72%)
Increased	6 (19%)	3 (7%)
**Choose correct foods**		
Decreased	8 (19%)	15 (24%)
Same	24 (56%)	40 (63%)
Increased	11 (26%)	8 (13%)
**Keep healthy weight**		
Decreased	10 (23%)	24 (37%)
Same	21 (49%)	33 (51%)
Increased	12 (28%)	8 (12%)
**Examine feet**		
Decreased	5 (11%)	13 (19%)
Same	33 (75%)	52 (78%)
Increased	6 (14%)	2 (3%)
**Healthy eating pattern**		
Decreased	15 (34%)	23 (35%)
Same	21 (48%)	32 (48%)
Increased	8 (18%)	11 (17%)
**Physical activity**		
Decreased	18 (40%)	34 (51%)
Same	23 (51%)	24 (36%)
Increased	4 (9%)	9 (13%)
**Mental wellbeing**		
Decreased	18 (40%)	27 (40%)
Same	25 (56%)	31 (46%)
Increased	2 (4%)	9 (13%)

Note: This table is restricted to participants who reported living alone for the question on living circumstances.

Qualitative responses regarding what respondents perceive they need to improve their diabetes self-management are summarised in the main manuscript.

Supplementary file 2: support needs of people living with diabetes during COVID-19

1.4. Cancellation of clinical appointments

Have you had to cancel diabetes appointments and check-ups because of the pandemic?	All	Type 1	Type 2
No	372 (48.2%)	249 (46.7%)	118 (53.6%)
Yes	399 (51.8%)	284 (53.3%)	102 (46.4%)
	771 (100.0%)	533 (100.0%)	220 (100.0%)

Qualitative responses regarding the impact of the cancellation of appointments are summarised in the appendices.

1.5. Sources used for information, advice and support

Which of these resources have you used for guidance on how you should behave regarding social/physical distancing measures? (Tick all that apply)	All (n = 770)	%	Type 1 (n = 535)	%	Type 2 (n = 217)	%
News channels	557	72.3	384	71.8	158	72.8
Public Health and government website	386	50.1	286	53.5	91	41.9
Diabetes UK website	461	59.9	340	63.6	110	50.7
NHS website	386	50.1	282	52.7	100	46.1
Other website	52	6.8	36	6.7	12	5.5
Social media	219	28.4	156	29.2	56	25.8
GP, diabetes nurse, healthcare professional	261	33.9	185	34.6	68	31.3
Family	174	22.6	125	23.4	41	18.9
Friends	111	14.4	79	14.8	28	12.9
Employer	113	14.7	85	15.9	23	10.6
Diabetes support group	67	8.7	52	9.7	11	5.1
Other	12	1.6	7	1.3	4	1.8

Which one have you used the most? (Tick one)	All	%	Type 1	%	Type 2	%
News channels	352	46.1	229	43.2	114	52.8
Public Health and government website	96	12.6	66	12.5	30	13.9
Diabetes UK website	113	14.8	83	15.7	28	13.0
NHS website	51	6.7	35	6.6	15	6.9
Other website	11	1.4	7	1.3	3	1.4
Social media	30	3.9	26	4.9	3	1.4
Facebook	31	4.1	24	4.5	7	3.2
GP, diabetes nurse, healthcare professional	33	4.3	22	4.2	8	3.7
Family	22	2.9	17	3.2	4	1.9
Friends	3	0.4	2	0.4	1	0.5
Employer	13	1.7	11	2.1	2	0.9
Diabetes support group	3	0.4	3	0.6	0	0.0
Other	5	0.7	4	0.8	1	0.5
Not applicable	1	0.1	1	0.2	0	0.0

Which of these resources have you been using for guidance on general diabetes self-management since the start of the	All (n = 713)	%	Type 1 (n = 487)	%	Type 2 (n = 208)	%

Supplementary file 2: support needs of people living with diabetes during COVID-19

**pandemic? (Tick all that apply)**						
News channels	101	14.2	64	13.1	34	16.4
Public Health and government website	76	10.7	48	9.9	24	11.5
Diabetes UK website	347	48.7	240	49.3	99	47.6
NHS website	153	21.5	99	20.3	53	25.5
Other website	28	3.9	19	3.9	8	3.9
Twitter	37	5.2	33	6.8	3	1.4
Facebook	63	8.8	46	9.5	17	8.2
GP, diabetes nurse, healthcare professional	207	29.0	148	30.4	51	24.5
Family	57	8.0	42	8.6	14	6.7
Friends	26	3.7	20	4.1	6	2.9
Employer	9	1.3	6	1.2	3	1.4
Diabetes support group	48	6.7	43	8.8	4	1.9
Other	15	2.1	9	1.9	6	2.9

**Which of these resources do you use to obtain emotional support? (Tick all that apply)**	**All (n = 687)**	**%**	**Type 1 (n = 474)**	**%**	**Type 2 (n = 196)**	**%**
Diabetes UK website – online forum	55	8.0	31	6.5	23	11.7
Diabetes UK helpline	15	2.2	7	1.5	7	3.6
Social media communities	71	10.3	54	11.4	16	8.2
GP, diabetes nurse, healthcare professional	86	12.5	61	12.9	23	11.7
Family	473	68.9	326	68.8	134	68.4
Friends	350	51.0	256	54.0	86	43.9
Employer	39	5.7	23	4.9	16	8.2
Diabetes support group	32	4.7	28	5.9	3	1.5
Other	10	1.5	8	1.7	2	1.0

**Which means do you use to obtain advice/guidance/support from outside your household? (tick all that apply)**	**All (n = 638*)**	**Type 1 (n = 442)**	**Type 2 (n = 179)**
Telephone	124 (19.4%)	80 (18.1%)	43 (24.0%)
Computer	467 (73.2%)	319 (72.2%)	133 (74.3%)
Mobile phone	429 (67.2%)	315 (71.3%)	102 (57.0%)
Someone in the house	41 (6.4%)	25 (5.7%)	15 (8.4%)
Other	3 (0.5%)	2 (0.5%)	1 (0.6%)

*113 people said not applicable

<u>Questions specific to those respondents living alone</u>

**If you are living alone, are you receiving support from people outside your household?**	**All***	**Type 1**	**Type 2**
No	83 (50.6%)	51 (47.2%)	30 (58.8%)
Yes	81 (49.4%)	57 (52.8%)	21 (41.2%)
	164 (100.0%)	108 (100.0%)	51 (100.0%)

*588 people said NA

This table is restricted to participants who reported living alone for the question on living circumstances.

**If yes, who from?**	**All**

Supplementary file 2: support needs of people living with diabetes during COVID-19

	(n = 81)
Family	55 (67.9%)
Friends	54 (66.7%)
Neighbours	22 (27.2%)
Other	4 (4.9%)

Opinions on information, advice, and support received

In general, how difficult or easy has it been for you to obtain INFORMATION/ADVICE applicable to you on the following?	All	Type 1	Type 2
**Glucose control**			
Very difficult	34 (5.5%)	15 (3.5%)	17 (10.2%)
Difficult	89 (14.4%)	52 (12.0%)	34 (20.4%)
Moderate	168 (27.3%)	123 (28.3%)	41 (24.6%)
Easy	164 (26.6%)	125 (28.8%)	34 (20.4%)
Very easy	161 (26.1%)	119 (27.4%)	41 (24.6%)
**Diet**			
Very difficult	39 (6.2%)	18 (4.3%)	21 (10.7%)
Difficult	80 (12.8%)	46 (11.0%)	32 (16.3%)
Moderate	174 (27.8%)	121 (29.0%)	51 (26.0%)
Easy	173 (27.6%)	120 (28.8%)	45 (23.0%)
Very easy	160 (25.6%)	112 (26.9%)	47 (24.0%)
**Physical activity**			
Very difficult	47 (7.3%)	28 (6.4%)	19 (9.8%)
Difficult	85 (13.2%)	55 (12.6%)	27 (13.9%)
Moderate	163 (25.3%)	109 (25.1%)	50 (25.8%)
Easy	199 (30.9%)	133 (30.6%)	60 (30.9%)
Very easy	149 (23.2%)	110 (25.3%)	38 (19.6%)
**Medication**			
Very difficult	34 (5.2%)	21 (4.7%)	11 (5.8%)
Difficult	94 (14.4%)	57 (12.8%)	35 (18.3%)
Moderate	165 (25.3%)	110 (24.6%)	50 (26.2%)
Easy	193 (29.6%)	139 (31.1%)	51 (26.7%)
Very easy	167 (25.6%)	120 (26.8%)	44 (23.0%)
**Emotional wellbeing**			
Very difficult	84 (13.3%)	58 (13.5%)	25 (13.3%)
Difficult	132 (20.9%)	89 (20.7%)	40 (21.3%)
Moderate	197 (31.2%)	136 (31.7%)	58 (30.9%)
Easy	122 (19.3%)	79 (18.4%)	37 (19.7%)
Very easy	96 (15.2%)	67 (15.6%)	28 (14.9%)
**Diabetes management (if showing symptoms)**			
Very difficult	59 (15.7%)	40 (14.5%)	19 (20.2%)
Difficult	70 (18.7%)	47 (17.1%)	21 (22.3%)
Moderate	103 (27.5%)	77 (28.0%)	26 (27.7%)
Easy	79 (21.1%)	62 (22.5%)	15 (16.0%)
Very easy	64 (17.1%)	49 (17.8%)	13 (13.8%)
**Physical/social/physical distancing**			
Very difficult	62 (8.7%)	48 (9.7%)	13 (6.5%)
Difficult	104 (14.5%)	81 (16.3%)	20 (10.0%)

Supplementary file 2: support needs of people living with diabetes during COVID-19

Moderate	169 (23.6%)	117 (23.5%)	48 (23.9%)
Easy	191 (26.7%)	126 (25.4%)	59 (29.4%)
Very easy	190 (26.5%)	125 (25.2%)	61 (30.3%)

In general, how difficult or easy has it been for you to obtain SUPPORT applicable to you on the following?	All	Type 1	Type 2
**Glucose control**			
Very difficult	63 (12.0%)	37 (10.1%)	23 (15.8%)
Difficult	111 (21.1%)	69 (18.8%)	39 (26.7%)
Moderate	141 (26.8%)	102 (27.7%)	36 (24.7%)
Easy	113 (21.4%)	86 (23.4%)	25 (17.1%)
Very easy	99 (18.8%)	74 (20.1%)	23 (15.8%)
**Diet**			
Very difficult	57 (10.9%)	32 (9.3%)	24 (14.1%)
Difficult	109 (20.8%)	67 (19.5%)	40 (23.5%)
Moderate	144 (27.5%)	98 (28.6%)	42 (24.7%)
Easy	122 (23.3%)	80 (23.3%)	40 (23.5%)
Very easy	91 (17.4%)	66 (19.2%)	24 (14.1%)
**Physical activity**			
Very difficult	60 (11.1%)	40 (11.1%)	19 (11.3%)
Difficult	109 (20.2%)	67 (18.7%)	40 (23.8%)
Moderate	145 (26.9%)	96 (26.7%)	44 (26.2%)
Easy	127 (23.6%)	83 (23.1%)	41 (24.4%)
Very easy	98 (18.2%)	73 (20.3%)	24 (14.3%)
**Medication**			
Very difficult	55 (9.8%)	34 (9.0%)	19 (11.0%)
Difficult	102 (18.1%)	66 (17.5%)	35 (20.3%)
Moderate	147 (26.1%)	94 (24.9%)	48 (27.9%)
Easy	138 (24.5%)	100 (26.5%)	33 (19.2%)
Very easy	122 (21.6%)	83 (22.0%)	37 (21.5%)
**Emotional wellbeing**			
Very difficult	89 (16.0%)	61 (16.2%)	26 (15.5%)
Difficult	130 (23.4%)	96 (25.5%)	32 (19.0%)
Moderate	166 (29.9%)	103 (27.4%)	58 (34.5%)
Easy	97 (17.5%)	66 (17.6%)	30 (17.9%)
Very easy	73 (13.2%)	50 (13.3%)	22 (13.1%)
**Diabetes management (if showing symptoms)**			
Very difficult	57 (18.7%)	40 (18.1%)	16 (20.0%)
Difficult	58 (19.0%)	43 (19.5%)	13 (16.3%)
Moderate	84 (27.5%)	55 (24.9%)	29 (36.3%)
Easy	55 (18.0%)	47 (21.3%)	8 (10.0%)
Very easy	51 (16.7%)	36 (16.3%)	14 (17.5%)
**Physical/social distancing**			
Very difficult	67 (11.1%)	47 (11.5%)	17 (9.5%)
Difficult	101 (16.7%)	77 (18.8%)	21 (11.7%)
Moderate	165 (27.4%)	114 (27.9%)	48 (26.8%)
Easy	143 (23.7%)	98 (24.0%)	41 (22.9%)
Very easy	127 (21.1%)	73 (17.8%)	52 (29.1%)
**How would you rate the QUALITY of the information/advice/support from the following sources or**	**All**	**Type 1**	**Type 2**

Supplementary file 2: support needs of people living with diabetes during COVID-19

channels?			
**Government**			
Very poor	105 (14.5%)	73 (14.3%)	28 (14.1%)
Poor	178 (24.5%)	140 (27.5%)	35 (17.6%)
Average	209 (28.8%)	137 (26.9%)	67 (33.7%)
Good	158 (21.8%)	109 (21.4%)	46 (23.1%)
Very good	76 (10.5%)	50 (9.8%)	23 (11.6%)
**Diabetes UK**			
Very poor	14 (2.2%)	10 (2.2%)	4 (2.2%)
Poor	34 (5.2%)	23 (5.0%)	10 (5.6%)
Average	152 (23.4%)	110 (24.0%)	38 (21.3%)
Good	293 (45.1%)	212 (46.3%)	76 (42.7%)
Very good	157 (24.2%)	103 (22.5%)	50 (28.1%)
**Social media**			
Very poor	56 (9.0%)	41 (9.5%)	15 (8.6%)
Poor	150 (24.1%)	101 (23.3%)	43 (24.7%)
Average	221 (35.5%)	152 (35.1%)	63 (36.2%)
Good	143 (23.0%)	101 (23.3%)	40 (23.0%)
Very good	52 (8.4%)	38 (8.8%)	13 (7.5%)
**News channels**			
Very poor	61 (8.5%)	41 (8.2%)	18 (9.0%)
Poor	151 (21.1%)	111 (22.3%)	38 (19.1%)
Average	234 (32.7%)	162 (32.5%)	65 (32.7%)
Good	214 (29.9%)	147 (29.5%)	61 (30.7%)
Very good	55 (7.7%)	37 (7.4%)	17 (8.5%)
**Friends**			
Very poor	29 (4.9%)	19 (4.5%)	10 (6.3%)
Poor	72 (12.1%)	49 (11.6%)	21 (13.3%)
Average	229 (38.4%)	160 (38.0%)	63 (39.9%)
Good	199 (33.4%)	145 (34.4%)	46 (29.1%)
Very good	67 (11.2%)	48 (11.4%)	18 (11.4%)
**Family**			
Very poor	23 (3.6%)	14 (3.1%)	8 (4.6%)
Poor	63 (9.9%)	43 (9.7%)	18 (10.3%)
Average	206 (32.3%)	146 (32.8%)	54 (31.0%)
Good	229 (35.9%)	162 (36.4%)	60 (34.5%)
Very good	116 (18.2%)	80 (18.0%)	34 (19.5%)
**Employer**			
Very poor	80 (17.8%)	56 (16.5%)	22 (22.4%)
Poor	65 (14.4%)	48 (14.1%)	15 (15.3%)
Average	113 (25.1%)	79 (23.2%)	30 (30.6%)
Good	121 (26.9%)	99 (29.1%)	19 (19.4%)
Very good	71 (15.8%)	58 (17.1%)	12 (12.2%)
**Healthcare team**			
Very poor	108 (18.0%)	71 (16.9%)	36 (22.2%)
Poor	115 (19.2%)	77 (18.3%)	34 (21.0%)
Average	116 (19.4%)	83 (19.7%)	30 (18.5%)
Good	140 (23.4%)	101 (24.0%)	35 (21.6%)
Very good	120 (20.0%)	89 (21.1%)	27 (16.7%)

Supplementary file 2: support needs of people living with diabetes during COVID-19

Nation/Region of England	n	Healthcare rating good or very good	%
Scotland	65	26	40.0
Wales	28	13	46.4
East England	45	20	44.4
East Midlands	32	13	40.6
Greater London	47	20	42.6
North East	19	4	21.1
North West	51	21	41.2
Northern Ireland	13	5	38.5
South East	132	68	51.5
South West	114	55	48.3
West Midlands	47	14	29.8

Qualitative responses regarding respondents' views on way to improve the information, advice and support from the sources above are summarised in the manuscript appendices.

Personal support networks

**For the next questions, please consider your current network of family, friends, contacts.**	**All**	**Living with others**	**Living alone**
**How would you rate their understanding of your CURRENT diabetes self-management needs?**			
Very poor	34 (4.5%)	25 (3.9%)	9 (7.9%)
Poor	98 (13.0%)	82 (12.8%)	16 (14.0%)
Average	243 (32.1%)	198 (30.8%)	45 (39.5%)
Good	225 (29.7%)	196 (30.5%)	29 (25.4%)
Very good	157 (20.7%)	142 (22.1%)	15 (13.2%)
**How would you rate their support in your diabetes self-management during the pandemic?**			
Very poor	37 (5.1%)	29 (4.6%)	8 (7.5%)
Poor	76 (10.4%)	64 (10.2%)	12 (11.2%)
Average	181 (24.7%)	148 (23.6%)	33 (30.8%)
Good	240 (32.7%)	204 (32.6%)	36 (33.6%)
Very good	199 (27.2%)	181 (28.9%)	18 (16.8%)

**How has the support you are receiving changed since before the pandemic? (0 = stayed the same)**	**All**	**Type 1**	**Type 2**
Decreased	144 (18.8%)	92 (17.4%)	49 (22.6%)
Same	405 (53.0%)	284 (53.7%)	116 (53.5%)
Increased	215 (28.1%)	153 (28.9%)	52 (24.0%)
	764 (100.0%)	529 (100.0%)	217 (100.0%)

Note: score given on a Likert scale ranging from -5 (Decreased) to 5 (Increased).

**In what ways do they CURRENTLY support**	**All**	**%**	**Type 1**	**%**	**Type 2**	**%**

Supplementary file 2: support needs of people living with diabetes during COVID-19

**your diabetes self-management? (Tick all that apply)**	**(n = 603*)**		**(n = 309)**		**(n = 119)**	
Food shopping/preparation	305	50.6	212	50.1	85	52.2
Picking up medication	292	48.4	215	50.8	69	42.3
Essential travel	84	13.9	61	14.4	19	11.7
Monitoring blood glucose	82	13.6	59	14.0	21	12.9
Emotional support	374	62.0	267	63.1	98	60.1
Access to online resources	65	10.8	42	9.9	21	12.9
Prompting self-management behaviours	174	28.9	121	28.6	48	29.5
Other	26	4.3	17	4.0	4	2.5

* 141 people said not applicable

2. Responses from parents, carers and partners of people living with diabetes

2.1. Geographical distribution of responses

**Region/nation**	**n**	**%**
Scotland	24	30.4
Wales	1	1.3
Channel Islands	1	1.3
East Midlands	1	1.3
East of England	6	7.6
Greater London	7	8.9
North East	4	5.1
North West	10	12.7
Northern Ireland	2	2.5
South East	5	6.3
South West	15	19.0
West Midlands	3	3.8

2.2. Demographic characteristics

	**All (n=79)**
**Gender, n (%)**	
Female	72 (91%)
Male	7 (9%)
**Age, mean (SD)**	45.2 (10.1)
**Ethnicity, n (%)**	
Asian or Asian British: Pakistani	1 (1%)
Black or Black British: Caribbean	1 (1%)
Other Black background	1 (1%)
Other ethnic group	1 (1%)
Other White background	2 (3%)
White: British	69 (87%)
White: Irish	4 (5%)

Living Circumstances

Sauchelli S, *et al. BMJ Open Diab Res Care* 2021; 9:e002162. doi: 10.1136/bmjdrc-2021-002162

Supplementary file 2: support needs of people living with diabetes during COVID-19

Are you currently living with the person who has diabetes?	n (%)
No	7 (9%)
Yes	72 (91%)
	79 (100%)

Has the number of people you are living with changed as a result of the coronavirus pandemic?	n (%)
No	74 (94%)
Yes	5 (6%)
	79 (100%)

Circumstances in relation to COVID-19

Have you been diagnosed with or displayed symptoms of coronavirus since the beginning of February?	n (%)
No	67 (85%)
Yes	6 (8%)
Not sure	6 (8%)
	79 (100%)

Which of the following best describes your current circumstances?	n (%)
Following stringent Physical/social/physical distancing	51 (65%)
Self-isolating at home	3 (4%)
Shielding group	4 (5%)
Shielding (but not in shielding group)	0 (0%)
Key worker/still leaving home to work	19 (24%)
Minimising interactions to protect someone in household	2 (3%)
	79 (100%)

2.3. Confidence in the ability to support diabetes self-management

**BEFORE** the coronavirus pandemic and social/physical distancing guidance I was confident that…	N	Median (IQR)
Check blood glucose	64	10 (10, 10)
Correct high blood glucose	68	10 (8, 10)
Correct low blood glucose	66	10 (10, 10)
Good blood glucose regulation	58	10 (8, 10)
Choose correct foods	75	10 (7, 10)
Keep healthy weight	72	8 (5, 10)
Examine feet	78	10 (5, 10)

Supplementary file 2: support needs of people living with diabetes during COVID-19

Healthy eating pattern	78	8 (6, 10)
Physical activity	78	8 (6, 10)
Mental wellbeing	78	8 (6, 9)

Note: score given on a Likert scale ranging from 0 (Could not do at all) to 10 (Certain could do). Not applicable was also an option to account inter-individual variability in condition and self-management requirements.

**AT PRESENT**, I am confident that…	**N**	**Median (IQR)**
Check blood glucose	68	10 (10, 10)
Correct high blood glucose	70	10 (8, 10)
Correct low blood glucose	67	10 (9, 10)
Good blood glucose regulation	57	10 (8, 10)
Choose correct foods	75	10 (8, 10)
Keep healthy weight	72	8 (5, 10)
Examine feet	78	10 (5, 10)
Healthy eating pattern	78	8 (6, 10)
Physical activity	78	8 (4, 10)
Mental wellbeing	78	8 (6, 9)

Note: score given on a Likert scale ranging from 0 (Could not do at all) to 10 (Certain could do). Not applicable was also an option to account inter-individual variability in condition and self-management requirements.

**Change in score**	**All**
**Check blood glucose**	
Decreased	4 (6%)
Same	55 (87%)
Increased	4 (6%)
**Correct high blood glucose**	
Decreased	6 (9%)
Same	50 (76%)
Increased	10 (15%)
**Correct low blood glucose**	
Decreased	9 (14%)
Same	51 (78%)
Increased	5 (8%)
**Good blood glucose regulation**	
Decreased	7 (13%)
Same	38 (68%)
Increased	11 (20%)
**Choose correct foods**	
Decreased	16 (22%)
Same	40 (54%)
Increased	18 (24%)
**Keep healthy weight**	
Decreased	15 (22%)
Same	39 (57%)
Increased	15 (22%)
**Examine feet**	

Sauchelli S, *et al. BMJ Open Diab Res Care* 2021; 9:e002162. doi: 10.1136/bmjdrc-2021-002162

Supplementary file 2: support needs of people living with diabetes during COVID-19

Decreased	7 (9%)
Same	63 (82%)
Increased	7 (9%)
**Healthy eating pattern**	
Decreased	20 (26%)
Same	45 (58%)
Increased	12 (16%)
**Physical activity**	
Decreased	21 (27%)
Same	49 (64%)
Increased	7 (9%)
**Mental wellbeing**	
Decreased	20 (26%)
Same	48 (62%)
Increased	9 (12%)

2.4. Sources used for information, advice and support

**Which of these resources have you used for guidance on how you should behave regarding social/physical distancing measures in relation to the person with diabetes? (Tick all that apply)**	**n (All = 78)**	**%**
News channels	47	60
Public Health and government website	33	42
Diabetes UK website	47	60
NHS website	47	60
Other website	3	4
Twitter	8	10
Facebook	19	24
GP, diabetes nurse, healthcare professional	31	40
Family	19	24
Friends	8	10
Employer	5	6
Diabetes support group	12	15
Other	5	6

**Which one have you use the most? (Tick one)**	**n**	**%**
News channels	26	34
Public Health and government website	9	12
Diabetes UK website	8	10
NHS website	10	13
Other website	0	0
Twitter	2	3
Facebook	4	5
GP, diabetes nurse, healthcare professional	9	12
Family	3	4
Friends	0	0
Employer	1	1
Diabetes support group	4	5
Other	0	0

Supplementary file 2: support needs of people living with diabetes during COVID-19

	77	100

**Which of these resources have you been using for guidance on general diabetes management since the start of the pandemic? (Tick all that apply)**	**n (All = 75)**	**%**
News channels	19	25
Public Health and government website	11	15
Diabetes UK website	37	49
NHS website	26	35
Other website	3	4
Twitter	2	3
Facebook	12	16
GP, diabetes nurse, healthcare professional	24	32
Family	5	7
Friends	1	1
Employer	1	1
Diabetes support group	11	15
Other	1	1

**Which of these resources do you use to obtain emotional support? (Tick all that apply)**	**n (All = 70)**	**%**
Diabetes UK website – online forum	6	9
Diabetes UK helpline	1	1
Facebook groups	12	17
GP, diabetes nurse, healthcare professional	12	17
Family	42	60
Friends	25	36
Neighbour	0	0
Employer	1	1
Diabetes support group	7	10
Other	2	3

**Which means do you use to obtain advice/guidance/support from outside your household? (tick all that apply)**	**All (n = 70*)**	**%**
Telephone	23	33
Computer	39	56
Mobile phone	50	71
Someone in the house	8	11
Other	0	0

*7 people said not applicable

Supplementary file 2: support needs of people living with diabetes during COVID-19

2.5. Opinions on information, advice, and support received

In general, how difficult or easy has it been for you to obtain INFORMATION/ADVICE applicable to the person you are helping on the following?	n (%)
**Glucose control**	
Very difficult	4 (6%)
Difficult	12 (18%)
Moderate	17 (25%)
Easy	21 (31%)
Very easy	14 (21%)
**Diet**	
Very difficult	4 (6%)
Difficult	13 (19%)
Moderate	11 (16%)
Easy	24 (35%)
Very easy	16 (24%)
**Physical activity**	
Very difficult	2 (3%)
Difficult	13 (19%)
Moderate	14 (20%)
Easy	23 (33%)
Very easy	17 (25%)
**Medication**	
Very difficult	5 (7%)
Difficult	13 (18%)
Moderate	16 (23%)
Easy	22 (31%)
Very easy	15 (21%)
**Emotional wellbeing**	
Very difficult	12 (17%)
Difficult	12 (17%)
Moderate	19 (27%)
Easy	15 (21%)
Very easy	13 (18%)
**Diabetes management (if showing symptoms)**	
Very difficult	8 (21%)
Difficult	10 (26%)
Moderate	9 (24%)
Easy	3 (8%)
Very easy	8 (21%)
**Physical/social distancing**	
Very difficult	10 (14%)
Difficult	9 (12%)
Moderate	16 (22%)
Easy	23 (31%)
Very easy	16 (22%)

Sauchelli S, *et al. BMJ Open Diab Res Care* 2021; 9:e002162. doi: 10.1136/bmjdrc-2021-002162

Supplementary file 2: support needs of people living with diabetes during COVID-19

**In general, how difficult or easy has it been for you to obtain SUPPORT applicable to the person you are helping on the following?**	**n (%)**
**Glucose control**	
Very difficult	8 (13%)
Difficult	10 (16%)
Moderate	12 (19%)
Easy	16 (26%)
Very easy	16 (26%)
**Diet**	
Very difficult	7 (11%)
Difficult	7 (11%)
Moderate	15 (24%)
Easy	19 (30%)
Very easy	15 (24%)
**Physical activity**	
Very difficult	6 (10%)
Difficult	10 (16%)
Moderate	13 (21%)
Easy	21 (33%)
Very easy	13 (21%)
**Medication**	
Very difficult	7 (11%)
Difficult	7 (11%)
Moderate	13 (20%)
Easy	20 (31%)
Very easy	17 (27%)
**Emotional wellbeing**	
Very difficult	16 (25%)
Difficult	6 (9%)
Moderate	17 (26%)
Easy	13 (20%)
Very easy	13 (20%)
**Diabetes management (if showing symptoms)**	
Very difficult	9 (26%)
Difficult	5 (15%)
Moderate	8 (24%)
Easy	4 (12%)
Very easy	8 (24%)
**Physical/social distancing**	
Very difficult	10 (16%)
Difficult	10 (16%)
Moderate	13 (20%)
Easy	16 (25%)
Very easy	15 (23%)

Supplementary file 2: support needs of people living with diabetes during COVID-19

How would you rate the QUALITY of the information/advice/support from the following sources or channels?	n (%)
**Government**	
Very poor	11 (14%)
Poor	15 (19%)
Average	23 (30%)
Good	19 (25%)
Very good	9 (12%)
**Diabetes UK**	
Very poor	1 (1%)
Poor	3 (4%)
Average	13 (19%)
Good	29 (42%)
Very good	23 (33%)
**Social media**	
Very poor	7 (10%)
Poor	9 (13%)
Average	29 (43%)
Good	18 (26%)
Very good	5 (7%)
**News channels**	
Very poor	8 (11%)
Poor	16 (23%)
Average	24 (34%)
Good	14 (20%)
Very good	9 (13%)
**Friends**	
Very poor	4 (7%)
Poor	7 (12%)
Average	22 (38%)
Good	19 (33%)
Very good	6 (10%)
**Family**	
Very poor	4 (6%)
Poor	8 (13%)
Average	22 (34%)
Good	20 (31%)
Very good	10 (16%)
**Employer**	
Very poor	8 (18%)
Poor	10 (23%)
Average	16 (36%)
Good	8 (18%)
Very good	2 (5%)
**Healthcare team**	
Very poor	3 (5%)
Poor	10 (15%)
Average	13 (20%)
Good	14 (22%)

Supplementary file 2: support needs of people living with diabetes during COVID-19

Very good	25 (38%)

2.6. Own role in supporting someone with diabetes

How would you rate your understanding of their CURRENT diabetes self-management needs?	n (%)
Very poor	1 (1%)
Poor	3 (4%)
Average	11 (14%)
Good	26 (33%)
Very good	37 (47%)

In what ways do you CURRENTLY support the individual in their diabetes self-management? (Tick all that apply)	n = 78*	%
Food shopping/preparation	69	88
Picking up medication	67	86
Essential travel	49	63
Monitoring blood glucose	49	63
Emotional support	69	88
Access to online resources	41	53
Prompting self-management behaviours	69	88
Other	6	8

*1 person said not applicable
