# Supplementary material for: Exploring support needs of people living with diabetes during the coronavirus COVID-19 pandemic: insights from a UK survey
Source: BMJ Open Diabetes Res Care. 2021 Jun 7;9(1):e002162. doi: 10.1136/bmjdrc-2021-002162 (PMC8186741; doi:10.1136/bmjdrc-2021-002162)
Supplement: Supplementary data [file bmjdrc-2021-002162supp003.pdf]

Supplementary File 3: support needs of people living with diabetes during COVID-19

Supplementary File 3

Title: Addressing support needs of people living with diabetes during the coronavirus COVID-19 pandemic: insights from a UK survey.

Sarah Sauchelli*[1], Julia Bradley[1], Clare England[1], Aidan Searle[1], Alex Whitmarsh[1]

[1] *National Institute for Health Research Bristol Biomedical Research Centre, University Hospitals of Bristol and Weston NHS Foundation Trust and University of Bristol.*

Three timepoints were identified in responses (24th of June, 20th of July, and 17th of August). Response times matched (± 2 days) major recruitment efforts but could not be linked to changes in government guidelines. Sample sizes were not sufficiently large to compare data across these time points but the data can be seen in Supplementary File 3. Here, survey responses are presented split across the three recruitment peaks.

Responses to the survey

1. Responses from people living with diabetes

1.1. Geographical distribution of responses

	Before July		July		August	
**Region/Nation**	n	%	n	%	n	%
Scotland	50	17.9	28	8.8	11	6.6
Wales	3	1.1	23	7.2	6	3.6
East Midlands	1	0.4	18	5.6	12	7.2
East of England	7	2.5	30	9.4	15	9.0
Greater London	10	3.6	37	11.6	18	10.8
North East	2	0.7	7	2.2	5	3.0
North West	3	1.1	34	10.6	18	10.8
Northern Ireland	2	0.7	7	2.2	5	3.0
South East	132	47.3	37	11.6	24	14.5
South West	60	21.5	52	16.3	30	18.1
West Midlands	8	2.9	34	10.6	15	9.0
Yorkshire & Humber	1	0.4	13	4.1	7	4.2
**Total**	279	100.0	320	100.0	166	100.0

1.2. Demographic characteristics.

	Before July		July		August	
**Diabetes group**	n	%	n	%	n	%
Type 1 diabetes	196	69.8	228	70.4	111	66.1
Type 2 diabetes	77	27.4	90	27.8	53	31.5
Another type of diabetes	8	2.8	6	1.9	4	2.4
	281	100.0	324	100.0	168	100.0

Supplementary File 3: support needs of people living with diabetes during COVID-19

Supplementary File 3: support needs of people living with diabetes during COVID-19

	Before July (n=281)	July (n=324)	August (n=168)
**Gender, n (%)**			
Female	162 (57.9%)	235 (73.2%)	119 (70.8%)
Male	115 (41.1%)	85 (26.5%)	49 (29.2%)
Other	3 (1.1%)	1 (0.3%)	0 (0.0%)
**Age, mean (SD)**	50.2 (14.7)	45.2 (14.2)	49.5 (14.0)
**Ethnicity, n (%)**			
Arab	1 (0.4%)	0 (0.0%)	0 (0.0%)
Asian or Asian British: Chinese	1 (0.4%)	1 (0.3%)	1 (0.6%)
Asian or Asian British: Indian	2 (0.7%)	4 (1.2%)	2 (1.2%)
Asian or Asian British: Pakistani	0 (0.0%)	0 (0.0%)	1 (0.6%)
Black or Black British: Caribbean	1 (0.4%)	2 (0.6%)	1 (0.6%)
Mixed: White and Asian	4 (1.4%)	1 (0.3%)	0 (0.0%)
Mixed: White and Black African	0 (0.0%)	1 (0.3%)	0 (0.0%)
Mixed: White and Black Caribbean	0 (0.0%)	1 (0.3%)	0 (0.0%)
Other ethnic group	0 (0.0%)	0 (0.0%)	1 (0.6%)
Other Mixed background	14 (5.0%)	13 (4.0%)	4 (2.4%)
Other White background	0 (0.0%)	1 (0.3%)	0 (0.0%)
Prefer not to answer	3 (1.1%)	0 (0.0%)	0 (0.0%)
White: British	251 (89.6%)	286 (89.1%)	156 (92.9%)
White: Irish	3 (1.1%)	11 (3.4%)	2 (1.2%)

Living circumstances

**Are you currently living alone?**	Before July	July	August
No	227 (81.1%)	277 (85.5%)	145 (86.3%)
Yes	53 (18.9%)	47 (14.5%)	23 (13.7%)
	280 (100.0%)	324 (100.0%)	168 (100.0%)

**Has the number of people you are living with changed as a result of the coronavirus pandemic?**	Before July	July	August
No	248 (88.3%)	288 (89.4%)	148 (89.2%)
Yes	33 (11.7%)	34 (10.6%)	18 (10.8%)
	281 (100.0%)	322 (100.0%)	166 (100.0%)

Supplementary File 3: support needs of people living with diabetes during COVID-19

Circumstances in relation to COVID-19

**Have you been diagnosed with or displayed symptoms of coronavirus since the beginning of February?**	**Before July**	**July**	**August**
No	232 (83.2%)	255 (78.9%)	136 (81.4%)
Yes	22 (7.9%)	30 (9.3%)	18 (10.8%)
Diagnosed with coronavirus	0 (0.0%)	1 (0.3%)	1 (0.6%)
Not sure	25 (9.0%)	37 (11.5%)	12 (7.2%)
	279 (100.0%)	323 (100.0%)	167 (100.0%)

**Which of the following best describes your current circumstances?**	**Before July**	**July**	**August**
Following stringent Physical/social/physical distancing	174 (61.9%)	215 (67.0%)	124 (74.7%)
Self-isolating at home	12 (4.3%)	1 (0.3%)	3 (1.8%)
Shielding group	22 (7.8%)	27 (8.4%)	10 (6.0%)
Shielding (but not in shielding group)	39 (13.9%)	31 (9.7%)	5 (3.0%)
Key worker/still leaving home to work	30 (10.7%)	44 (13.7%)	23 (13.9%)
Other	3 (1.1%)	0 (0.0%)	1 (0.6%)
Don't know	1 (0.4%)	3 (0.9%)	0 (0.0%)
	281 (100.0%)	321 (100.0%)	166 (100.0%)

1.3. Confidence in diabetes self-management

**BEFORE** the coronavirus pandemic and social/physical distancing guidance I was confident that I was able to…	**Before July**		**July**		**August**	
	n	Median (IQR)	n	Median (IQR)	n	Median (IQR)
Check blood glucose	259	10 (10, 10)	302	10 (10, 10)	159	10 (10, 10)
Correct high blood glucose	258	10 (8, 10)	293	10 (8, 10)	152	10 (8, 10)
Correct low blood glucose	250	10 (9, 10)	285	10 (9, 10)	145	10 (10, 10)
Good blood glucose regulation	207	10 (8, 10)	233	9 (7, 10)	131	10 (7, 10)
Choose correct foods	262	10 (8, 10)	309	10 (7, 10)	158	10 (8, 10)
Keep healthy weight	274	8 (5, 10)	317	6 (4, 9)	164	8 (4, 10)
Examine feet	277	10 (9, 10)	324	10 (8, 10)	168	10 (8, 10)
Healthy eating pattern	278	9 (7, 10)	324	8 (6, 10)	168	8 (6, 10)
Physical activity	278	8 (6, 10)	324	7 (5, 10)	168	8 (5, 10)
Mental wellbeing	279	8 (6, 10)	324	7 (5, 9)	167	8 (5, 10)

Note: score given on a Likert scale ranging from 0 (Could not do at all) to 10 (Certain could do). Not applicable was also an option to account inter-individual variability in condition and self-management requirements.

**AT PRESENT**, I am confident that…	**Before July**		**July**		**August**	
	n	Median (IQR)	n	Median (IQR)	n	Median (IQR)
Check blood glucose	264	10 (10, 10)	304	10 (10, 10)	159	10 (10, 10)
Correct high blood glucose	263	10 (8, 10)	302	9 (8, 10)	153	10 (8, 10)
Correct low blood glucose	258	10 (9, 10)	288	10 (9, 10)	151	10 (9, 10)
Good blood glucose	211	10 (8, 10)	234	9 (7, 10)	133	10 (7, 10)

Sauchelli S, *et al. BMJ Open Diab Res Care* 2021; 9:e002162. doi: 10.1136/bmjdrc-2021-002162

Supplementary File 3: support needs of people living with diabetes during COVID-19

regulation						
Choose correct foods	267	10 (8, 10)	315	9 (7, 10)	162	10 (8, 10)
Keep healthy weight	277	7 (5, 10)	318	6 (3, 9)	164	7 (4, 10)
Examine feet	279	10 (8, 10)	318	10 (8, 10)	167	10 (8, 10)
Healthy eating pattern	278	8 (7, 10)	321	8 (5, 10)	167	8 (7, 10)
Physical activity	279	8 (5, 10)	322	6 (4, 9)	167	8 (5, 10)
Mental wellbeing	277	8 (5, 10)	322	6 (4, 8)	166	8 (5, 10)

Note: score given on a Likert scale ranging from 0 (Could not do at all) to 10 (Certain could do). Not applicable was also an option to account inter-individual variability in condition and self-management requirements.

**Change in score**	**Before July**	**July**	**August**
**Check blood glucose**			
Decreased	14 (5.4%)	38 (12.8%)	16 (10.2%)
Same	225 (87.5%)	247 (82.9%)	128 (81.5%)
Increased	18 (7.0%)	13 (4.4%)	13 (8.3%)
**Correct high blood glucose**			
Decreased	30 (11.7%)	51 (17.6%)	14 (9.3%)
Same	205 (79.8%)	191 (65.9%)	114 (75.5%)
Increased	22 (8.6%)	48 (16.6%)	23 (15.2%)
**Correct low blood glucose**			
Decreased	29 (11.7%)	47 (16.7%)	16 (11.1%)
Same	198 (80.2%)	208 (74.0%)	116 (80.6%)
Increased	20 (8.1%)	26 (9.3%)	12 (8.3%)
**Good blood glucose regulation**			
Decreased	22 (11.0%)	50 (22.0%)	20 (15.6%)
Same	155 (77.5%)	139 (61.2%)	86 (67.2%)
Increased	23 (11.5%)	38 (16.7%)	22 (17.2%)
**Choose correct foods**			
Decreased	54 (21.1%)	83 (27.1%)	36 (22.9%)
Same	170 (66.4%)	174 (56.9%)	96 (61.1%)
Increased	32 (12.5%)	49 (16.0%)	25 (15.9%)
**Keep healthy weight**			
Decreased	69 (25.5%)	96 (30.8%)	37 (22.8%)
Same	150 (55.4%)	155 (49.7%)	81 (50.0%)
Increased	52 (19.2%)	61 (19.6%)	44 (27.2%)
**Examine feet**			
Decreased	27 (9.8%)	49 (15.4%)	11 (6.6%)
Same	223 (81.1%)	239 (75.2%)	136 (81.4%)
Increased	25 (9.1%)	30 (9.4%)	20 (12.0%)
**Healthy eating pattern**			
Decreased	78 (28.4%)	103 (32.1%)	45 (26.9%)
Same	157 (57.1%)	151 (47.0%)	85 (50.9%)
Increased	40 (14.5%)	67 (20.9%)	37 (22.2%)
**Physical activity**			
Decreased	87 (31.5%)	114 (35.4%)	44 (26.3%)
Same	145 (52.5%)	139 (43.2%)	77 (46.1%)
Increased	44 (15.9%)	69 (21.4%)	46 (27.5%)
**Mental wellbeing**			

Sauchelli S, *et al. BMJ Open Diab Res Care* 2021; 9:e002162. doi: 10.1136/bmjdrc-2021-002162

Supplementary File 3: support needs of people living with diabetes during COVID-19

Decreased	92 (33.5%)	148 (46.0%)	42 (25.5%)
Same	157 (57.1%)	121 (37.6%)	84 (50.9%)
Increased	26 (9.5%)	53 (16.5%)	39 (23.6%)

1.4. Cancellation of clinical appointments

Have you had to cancel diabetes appointments and check-ups because of the pandemic?	Before July	July	August
No	143 (50.9%)	135 (41.9%)	94 (56.0%)
Yes	138 (49.1%)	187 (58.1%)	74 (44.0%)
	281 (100.0%)	322 (100.0%)	168 (100.0%)

Qualitative responses regarding the impact of the cancellation of appointments are summarised in the appendices.

1.5. Sources used for information, advice and support

Which of these resources have you used for guidance on how you should behave regarding social/physical distancing measures? (Tick all that apply)	Before July (n = 279)	%	July (n = 324)	%	August (n = 167)	%
News channels	217	77.8	216	66.7	124	74.3
Public Health and government website	125	44.8	182	56.2	79	47.3
Diabetes UK website	134	48.0	217	67.0	110	65.9
NHS website	137	49.1	175	54.0	74	44.3
Other website	13	4.7	27	8.3	12	7.2
Social media	59	21.2	113	34.9	47	28.1
GP, diabetes nurse, healthcare professional	102	36.6	107	33.0	52	31.1
Family	67	24.0	78	24.1	29	17.4
Friends	44	15.8	47	14.5	20	12.0
Employer	45	16.1	48	14.8	20	12.0
Diabetes support group	16	5.7	36	11.1	16	9.0
Other	3	1.1	6	1.9	3	1.8

Which one have you used the most? (Tick one)	Before July	%	July	%	August	%
News channels	149	53.8	130	40.5	73	44.0
Public Health and government website	28	10.1	44	13.7	24	14.5
Diabetes UK website	30	10.8	56	17.5	28	16.9
NHS website	20	8.2	18	5.6	13	7.8
Other website	4	1.4	4	1.3	2	1.2
Social media	11	4.0	15	4.7	4	2.4
Facebook	5	1.8	7	2.2	1	0.6
GP, diabetes nurse, healthcare professional	12	4.3	13	4.4	7	4.2
Family	8	2.9	10	3.1	4	2.4
Friends	1	0.4	1	0.3	1	0.6
Employer	5	1.8	18	5.6	8	4.8
Diabetes support group	1	0.4	2	0.6	0	0.0
Other	2	0.7	2	0.6	1	0.6

Sauchelli S, *et al. BMJ Open Diab Res Care* 2021; 9:e002162. doi: 10.1136/bmjdrc-2021-002162

Supplementary File 3: support needs of people living with diabetes during COVID-19

Not applicable	1	0.4	0	0.0	0	0.0

**Which of these resources have you been using for guidance on general diabetes self-management since the start of the pandemic? (Tick all that apply)**	**Before July (n = 252)**	**%**	**July (n = 304)**	**%**	**August (n = 157)**	**%**
News channels	44	17.5	31	10.2	26	16.6
Public Health and government website	25	9.9	33	10.9	18	11.5
Diabetes UK website	112	44.4	157	51.6	78	49.7
NHS website	60	23.8	62	20.4	31	19.8
Other website	7	2.8	14	4.6	7	4.5
Twitter	9	3.6	21	6.9	7	4.5
Facebook	6	2.4	38	12.5	19	12.1
GP, diabetes nurse, healthcare professional	81	32.1	90	29.6	36	22.9
Family	23	9.1	27	8.9	7	4.5
Friends	10	4.0	13	4.3	3	1.9
Employer	4	1.6	3	1.0	2	1.2
Diabetes support group	11	4.4	27	8.9	10	6.4
Other	8	3.2	4	1.3	3	1.9

**Which of these resources do you use to obtain emotional support? (Tick all that apply)**	**Before July (n = 247)**	**%**	**July (n = 291)**	**%**	**August (n = 149)**	**%**
Diabetes UK website – online forum	19	7.7	21	7.2	15	10.1
Diabetes UK helpline	6	2.4	7	2.4	2	1.3
Social media communities	22	8.9	32	11.0	17	11.4
GP, diabetes nurse, healthcare professional	32	13.0	31	10.7	23	15.4
Family	175	70.9	194	66.7	104	69.8
Friends	126	51.0	153	52.6	71	47.7
Employer	16	6.5	20	6.9	3	2.0
Diabetes support group	11	4.5	16	5.5	5	3.4
Other	6	2.4	2	0.7	2	1.3

**Which means do you use to obtain advice/guidance/support from outside your household? (tick all that apply)**	**Before July (n = 219)**	**July (n = 273)**	**August (n = 146)**
Telephone	53 (24.2%)	47 (17.2%)	24 (16.4%)
Computer	171 (78.1%)	194 (71.1%)	102 (69.9%)
Mobile phone	43 (65.3%)	189 (69.2%)	97 (66.4%)
Someone in the house	19 (8.7%)	16 (5.9%)	6 (4.1%)
Other	0 (0.0%)	3 (1.1%)	0 (0.0%)

Questions specific to those respondents living alone

**If you are living alone, are you receiving support from people outside your household?**	**Before July**	**July**	**August**
No	36 (51.4%)	34 (51.5%)	13 (46.4%)

Supplementary File 3: support needs of people living with diabetes during COVID-19

Yes	34 (48.6%)	32 (48.5%)	15 (53.6%)
	70 (100.0%)	66 (100.0%)	28 (100.0%)

This table is restricted to participants who reported living alone for the question on living circumstances.

Opinions on information, advice, and support received

In general, how difficult or easy has it been for you to obtain INFORMATION/ADVICE applicable to you on the following?	Before July	July	August
**Glucose control**			
Very difficult	6 (2.9%)	21 (7.8%)	7 (5.0%)
Difficult	22 (10.6%)	49 (18.2%)	18 (12.9%)
Moderate	67 (32.4%)	70 (26.0%)	31 (22.1%)
Easy	57 (27.5%)	73 (27.1%)	34 (24.3%)
Very easy	55 (26.6%)	56 (20.8%)	50 (35.7%)
**Diet**			
Very difficult	10 (4.7%)	20 (7.2%)	9 (6.6%)
Difficult	18 (8.4%)	48 (17.4%)	14 (10.3%)
Moderate	67 (31.3%)	79 (28.6%)	28 (20.6%)
Easy	55 (25.7%)	76 (27.5%)	42 (30.9%)
Very easy	64 (29.9%)	53 (19.2%)	43 (31.6%)
**Physical activity**			
Very difficult	14 (6.3%)	24 (8.5%)	9 (6.5%)
Difficult	26 (11.7%)	45 (15.9%)	14 (10.1%)
Moderate	59 (26.6%)	75 (26.5%)	29 (21.0%)
Easy	66 (29.7%)	85 (30.0%)	48 (34.8%)
Very easy	57 (25.7%)	54 (19.1%)	38 (27.5%)
**Medication**			
Very difficult	3 (1.3%)	26 (9.0%)	5 (3.7%)
Difficult	27 (11.8%)	48 (16.6%)	19 (14.0%)
Moderate	60 (26.3%)	77 (26.6%)	28 (20.6%)
Easy	64 (28.1%)	87 (30.1%)	42 (30.9%)
Very easy	74 (32.5%)	51 (17.6%)	42 (30.9%)
**Emotional wellbeing**			
Very difficult	9 (4.4%)	57 (19.8%)	18 (12.9%)
Difficult	40 (19.7%)	65 (22.6%)	27 (19.3%)
Moderate	68 (33.5%)	83 (28.8%)	46 (32.9%)
Easy	43 (21.2%)	57 (19.8%)	22 (15.7%)
Very easy	43 (21.2%)	26 (9.0%)	27 (19.3%)
**Diabetes management (if showing symptoms)**			
Very difficult	18 (13.4%)	31 (19.9%)	10 (11.8%)
Difficult	28 (20.9%)	26 (16.7%)	16 (18.8%)
Moderate	36 (26.9%)	45 (28.8%)	22 (25.9%)
Easy	30 (22.4%)	32 (20.5%)	17 (20.0%)
Very easy	22 (16.4%)	22 (14.1%)	20 (23.5%)
**Physical/social/physical distancing**			
Very difficult	26 (10.2%)	30 (9.8%)	6 (3.9%)
Difficult	34 (13.3%)	59 (19.2%)	11 (7.1%)
Moderate	55 (21.6%)	70 (22.8%)	44 (28.6%)
Easy	66 (25.9%)	81 (26.4%)	44 (28.6%)

Supplementary File 3: support needs of people living with diabetes during COVID-19

Very easy	74 (29.0%)	67 (21.8%)	49 (31.8%)

**In general, how difficult or easy has it been for you to obtain SUPPORT applicable to you on the following?**	**Before July**	**July**	**August**
**Glucose control**			
Very difficult	15 (9.3%)	35 (13.9%)	13 (11.3%)
Difficult	29 (18.0%)	61 (24.3%)	21 (18.3%)
Moderate	48 (29.8%)	61 (24.3%)	32 (27.8%)
Easy	36 (22.4%)	55 (21.9%)	22 (19.1%)
Very easy	33 (20.5%)	39 (15.5%)	27 (23.5%)
**Diet**			
Very difficult	13 (8.0%)	31 (12.4%)	13 (11.8%)
Difficult	28 (17.2%)	64 (25.6%)	17 (15.5%)
Moderate	46 (28.2%)	66 (26.4%)	32 (29.1%)
Easy	45 (27.6%)	55 (22.0%)	22 (20.0%)
Very easy	31 (19.0%)	34 (13.6%)	26 (23.6%)
**Physical activity**			
Very difficult	17 (9.9%)	31 (12.2%)	12 (10.6%)
Difficult	30 (17.4%)	61 (24.0%)	18 (15.9%)
Moderate	44 (25.6%)	66 (26.0%)	35 (31.0%)
Easy	42 (24.4%)	62 (24.4%)	23 (20.4%)
Very easy	39 (22.7%)	34 (13.4%)	25 (22.1%)
**Medication**			
Very difficult	9 (4.9%)	35 (13.3%)	11 (9.3%)
Difficult	26 (14.2%)	54 (20.5%)	22 (18.6%)
Moderate	56 (30.6%)	60 (22.8%)	31 (26.3%)
Easy	41 (22.4%)	73 (27.8%)	24 (20.3%)
Very easy	51 (27.9%)	41 (15.6%)	30 (25.4%)
**Emotional wellbeing**			
Very difficult	16 (9.5%)	54 (20.0%)	19 (16.2%)
Difficult	35 (20.8%)	75 (27.8%)	20 (17.1%)
Moderate	48 (28.6%)	73 (27.0%)	45 (38.5%)
Easy	39 (23.2%)	43 (15.9%)	15 (12.8%)
Very easy	30 (17.9%)	25 (9.3%)	18 (15.4%)
**Diabetes management (if showing symptoms)**			
Very difficult	18 (17.3%)	30 (22.6%)	9 (13.2%)
Difficult	20 (19.2%)	27 (20.3%)	11 (16.2%)
Moderate	27 (26.0%)	34 (25.6%)	23 (33.8%)
Easy	22 (21.2%)	25 (18.8%)	8 (11.8%)
Very easy	17 (16.3%)	17 (12.8%)	17 (25.0%)
**Physical/social distancing**			
Very difficult	23 (11.6%)	34 (12.3%)	10 (7.8%)
Difficult	32 (16.2%)	57 (20.7%)	12 (9.3%)
Moderate	54 (27.3%)	70 (25.4%)	41 (31.8%)
Easy	42 (21.2%)	68 (24.6%)	33 (25.6%)
Very easy	47 (23.7%)	47 (17.0%)	33 (25.6%)
**How would you rate the QUALITY of the information/advice/support from the following sources or channels?**	**Before July**	**July**	**August**

Supplementary File 3: support needs of people living with diabetes during COVID-19

**Government**			
Very poor	38 (14.7%)	47 (15.0%)	20 (13.1%)
Poor	64 (24.7%)	83 (26.4%)	31 (20.3%)
Average	69 (26.6%)	94 (29.9%)	46 (30.1%)
Good	59 (22.8%)	62 (19.7%)	37 (24.2%)
Very good	29 (11.2%)	28 (8.9%)	19 (12.4%)
**Diabetes UK**			
Very poor	7 (3.3%)	6 (2.1%)	1 (0.7%)
Poor	21 (9.9%)	7 (2.4%)	6 (4.0%)
Average	41 (19.2%)	83 (29.0%)	28 (18.5%)
Good	97 (45.5%)	124 (43.4%)	72 (47.7%)
Very good	47 (22.1%)	66 (23.1%)	44 (29.1%)
**Social media**			
Very poor	19 (10.2%)	25 (8.6%)	12 (8.2%)
Poor	47 (25.3%)	74 (25.5%)	29 (19.9%)
Average	70 (37.6%)	104 (35.9%)	47 (32.2%)
Good	37 (19.9%)	67 (23.1%)	39 (26.7%)
Very good	13 (7.0%)	20 (6.9%)	19 (13.0%)
**News channels**			
Very poor	18 (7.0%)	33 (10.9%)	10 (6.5%)
Poor	47 (18.2%)	71 (23.4%)	33 (21.6%)
Average	80 (31.0%)	102 (33.6%)	52 (34.0%)
Good	90 (34.9%)	83 (27.3%)	41 (26.8%)
Very good	23 (8.9%)	15 (4.9%)	17 (11.1%)
**Friends**			
Very poor	7 (3.4%)	17 (6.5%)	5 (3.8%)
Poor	25 (12.3%)	34 (13.0%)	13 (9.8%)
Average	75 (36.9%)	113 (43.3%)	41 (31.1%)
Good	69 (34.0%)	77 (29.5%)	53 (40.2%)
Very good	27 (13.3%)	20 (7.7%)	20 (15.2%)
**Family**			
Very poor	6 (2.7%)	14 (5.0%)	3 (2.2%)
Poor	22 (10.0%)	29 (10.4%)	12 (8.8%)
Average	62 (28.1%)	103 (36.9%)	41 (29.9%)
Good	78 (35.3%)	100 (35.8%)	51 (37.2%)
Very good	53 (24.0%)	33 (11.8%)	30 (21.9%)
**Employer**			
Very poor	19 (12.8%)	47 (22.7%)	14 (14.9%)
Poor	25 (16.8%)	33 (15.9%)	7 (7.4%)
Average	33 (22.1%)	51 (24.6%)	29 (30.9%)
Good	42 (28.2%)	50 (24.2%)	29 (30.9%)
Very good	30 (20.1%)	26 (12.6%)	15 (16.0%)
**Healthcare team**			
Very poor	15 (7.6%)	65 (24.3%)	28 (21.1%)
Poor	45 (22.7%)	47 (17.5%)	23 (17.3%)
Average	38 (19.2%)	55 (20.5%)	23 (17.3%)
Good	56 (28.3%)	52 (19.4%)	32 (24.1%)
Very good	44 (22.2%)	49 (18.3%)	27 (20.3%)

Personal support networks

Supplementary File 3: support needs of people living with diabetes during COVID-19

For the next questions, please consider your current network of family, friends, contacts.	Before July	July	August
How would you rate their understanding of your CURRENT diabetes self-management needs?			
Very poor	10 (3.6%)	18 (5.6%)	6 (3.6%)
Poor	31 (11.4%)	48 (15.1%)	19 (11.5%)
Average	80 (29.3%)	107 (33.5%)	57 (34.3%)
Good	85 (31.1%)	96 (30.1%)	44 (26.5%)
Very good	67 (24.5%)	50 (15.7%)	40 (24.1%)
How would you rate their support in your diabetes self-management during the pandemic?			
Very poor	12 (4.7%)	21 (6.7%)	4 (2.4%)
Poor	19 (7.5%)	37 (11.8%)	20 (12.2%)
Average	54 (21.2%)	85 (27.0%)	42 (25.6%)
Good	88 (34.5%)	105 (33.3%)	47 (28.7%)
Very good	82 (32.2%)	67 (21.3%)	51 (31.1%)

How has the support you are receiving changed since before the pandemic? (0 = stayed the same)	Before July	July	August
Decreased	36 (12.9%)	76 (23.7%)	32 (19.4%)
Same	157 (56.5%)	158 (49.2%)	90 (54.6%)
Increased	85 (30.6%)	87 (27.1%)	43 (26.1%)
	278 (100.0%)	321 (100.0%)	165 (100.0%)

Note: score given on a Likert scale ranging from -5 (Decreased) to 5 (Increased).

In what ways do they CURRENTLY support your diabetes self-management? (Tick all that apply)	Before July (n = 217)	%	July (n = 256)	%	August (n = 130)	%
Food shopping/preparation	122	56.2	132	51.6	51	39.2
Picking up medication	113	52.1	124	48.4	55	42.3
Essential travel	30	13.8	35	13.7	19	14.6
Monitoring blood glucose	27	12.4	36	14.1	19	14.6
Emotional support	138	63.6	151	59.0	85	65.4
Access to online resources	25	11.5	22	8.6	18	13.9
Prompting self-management behaviours	56	25.8	83	32.4	35	26.9
Other	11	5.1	14	5.5	1	0.8
